# Is neglect different? Social determinants of child protection plans by recorded maltreatment type in England: a longitudinal ecological study, 2014/2015–2022/2023

**DOI:** 10.1136/bmjopen-2026-119211

**Published:** 2026-08-05

**Authors:** Francesca Murley, Nurgul Gecer, Gianmaria Niccodemi, Michelle Black, Katie Fahy, David Taylor-Robinson, Philip McHale, Davara Bennett

**Affiliations:** 1 Department of Public Health, Policy and Systems, University of Liverpool, Liverpool, UK

**Keywords:** MENTAL HEALTH, Child protection, Primary Prevention, PUBLIC HEALTH, STATISTICS & RESEARCH METHODS, Longitudinal studies

## Abstract

**Abstract:**

**Objectives:**

Neglect is the leading cause of acute child protection interventions across high-income countries. In England, reducing the number of children subject to these interventions is a policy priority. However, limited evidence on the health and social factors driving recent trends in neglect constrains effective prevention. We aimed to assess the associations of area-level adult mental health, child poverty and employment, with recorded neglect, and to compare these associations with those observed for other forms of maltreatment.

**Design:**

Longitudinal ecological study using panel data.

**Setting:**

147 upper-tier local authorities in England from 2014/2015 to 2022/2023.

**Participants:**

Children aged 0–17 years.

**Outcome measures:**

Primary: annual rate of new child protection plans (CPPs) due to neglect. Secondary: annual rate of CPPs due to other maltreatment types (emotional, physical, sexual abuse) and rate of children entering care. We used two-way linear fixed effects models to estimate associations between each exposure and outcome, controlling for confounders.

**Results:**

Rising adult mental ill health was associated with higher rates of neglect and emotional abuse CPPs at area-level, but CIs included the null. Child poverty was positively associated with neglect CPPs: a one-percentage point increase was associated with around one additional CPP per 10 000 children (95% CI 0.2 to 2.2), equivalent to a model-estimated difference of over 30 000 plans over the study period. Higher employment was associated with fewer physical abuse CPPs, though CIs included the null. Care entry was positively associated with mental ill health and child poverty, and negatively associated with employment.

**Conclusions:**

Recorded neglect and care entry appear particularly sensitive to wider social conditions affecting caregiving capacity and service response. Preventative policies to support parental mental health and tackle child poverty may help reduce the need for the most common and acute child protection interventions.

**STRENGTHS AND LIMITATIONS OF THIS STUDY**

- Longitudinal ecological design exploiting within-local authority variation over time.
- Two-way fixed effects modelling controlling for time-invariant unobserved confounding.
- Counterfactual simulation methods to estimate population burden associated with exposure trends.
- Use of population-level administrative data reflecting both underlying need and service response.
- Ecological design limits inference to area-level associations.

## Introduction

 Neglect is the leading cause of children being taken into state care across high-income countries.^1^ In England and Wales, incidence rates of child protection plans (CPPs) due to neglect, implemented when children are deemed at risk of significant harm, have increased more than sixfold since 1990,^2 3^ placing increased pressure on local authority (LA) children’s services. In England, in 2024–2025, neglect accounted for almost half of new CPPs; emotional, physical and sexual abuse accounted for 37%, 8% and 4%, respectively.^3^ Safely reducing the number of children subject to statutory child protection interventions is now a central policy objective in England.^4^ Achieving this objective requires an understanding of the modifiable factors associated with variation in neglect.

The National Society for the Prevention of Cruelty to Children defines child neglect as the persistent failure to meet a child’s basic needs, including physical (food, clothing, shelter and supervision), educational (receipt of an education), emotional (nurture and stimulation) and medical (receipt of healthcare).^5^ As with child physical, emotional and sexual abuse, neglect is associated with long-term harms, including increased odds of depressive disorders, drug use and suicide attempts.^6^

However, critical scholarship cautions against treating different forms of maltreatment as equivalent. While abuse is characterised by acts of commission, neglect is generally defined by acts of omission—not what carers do, but what they fail to do, or what they are unable to provide. This distinction raises questions about capacity and intention. Neglect-related harm may be unintentional, arising from constrained caregiver capabilities linked to disadvantaged socioeconomic conditions and family stress.^7–9^ The social determinants may be especially relevant to understanding neglect, relative to other maltreatment types. A systematic review of social workers’ perceptions of neglect found widespread recognition of the contribution of parental mental health problems and addiction to neglect—though limited recognition of the role of poverty in structuring these factors.^10^

These complexities may also have implications for how neglect is identified and recorded. Measurement is a fundamental challenge in child maltreatment research.^11^ Definitions of abuse and neglect are neither fixed nor culturally neutral.^12^ Understandings of neglect in particular have evolved over time, shaped by societal expectations of good parenting.^13^ Within child welfare systems, a determination of child neglect also relies on policy frameworks, professional norms and judgement, intervention thresholds and data collection practices, all of which may vary within and between LAs, regions and countries. Administrative data are more likely to record forms of adversity that are more visible, disclosed or surveilled. Families experiencing poverty and poor mental health may be more likely to face both caregiving challenges and heightened scrutiny from services. Together, these processes may shape observed patterns of recorded neglect. There is a lack of clarity on whether social determinants of adversity are specifically associated with recorded neglect, or more generally with child protection involvement. This has implications for policy strategies to safely reduce rates of neglect.

Empirical research into the antecedents of different types of child maltreatment shows substantial overlap in risk factors by type.^14 15^ A recent systematic review and meta-analysis found that child neglect, physical and emotional abuse all shared the risk factors of parental mental ill health, young parental age and low income and economic status. Neglect, physical and sexual abuse were linked to parental substance misuse; neglect and emotional abuse to parental maltreatment history. There were also divergences. Neglect was not specifically associated with parental education, employment and family size—all risk factors for other maltreatment types.^16^ A separate systematic review focused on unemployment further complicates this picture, showing associations of all maltreatment types with paternal or any unemployment, and no associations of any kind with solely maternal unemployment.^17^ There is currently insufficient evidence to determine whether neglect is more sensitive to socioeconomic conditions than other maltreatment types, in part because different measures yield these different patterns of association.^18^ However, a 2023 study from the USA suggests that neighbourhood poverty may have a lasting effect on child neglect only—a relationship fully mediated by family monetary well-being.^19^

Overall, despite extensive child maltreatment research, there remain important gaps in our understanding of the determinants of neglect. The neglect-specific evidence base is dominated by studies from the USA, limiting transferability to the UK context.^14 15 17^ Longitudinal analyses remain relatively scarce, and few studies span the last decade—a period in the UK marked by austerity policies, tiered COVID-19 pandemic restrictions^20^ and the rising cost of living,^21^ each with potential implications for caregiving capacity and service response.^22^ In England, to our knowledge, no ecological studies have leveraged the availability of small-area population mental health indicators^23^ to assess associations with child protection outcomes. An ecological study assessed the contribution of child poverty to CPP-initiation, but did not disaggregate by abuse type and only spanned a 5-year period to March 2020, calling for updated analyses when further data were published.^24^

In this study, we aimed to fill these gaps. First, we assessed whether adult mental health was associated with CPPs due to recorded neglect, in English LAs, between 2014/2015 and 2022/2023, comparing results with other recorded maltreatment types and with care entry, a more acute intervention. Second, we assessed the associations of child poverty and employment with neglect and all other maltreatment types.

## Methods

We undertook a longitudinal, ecological study at the upper-tier LA level in England, using panel data from 147 LAs between 2014/2015 and 2022/2023. During this time, some LAs underwent boundary changes. Where local authorities split cleanly (Northamptonshire into North and West Northamptonshire from 2021/2022, Cumbria into Cumberland and Westmorland and Furness from 2023/2024), we harmonised the data, retaining the presplit boundary throughout the study period. We excluded two LAs due to their very small population size (City of London and Isles of Scilly), and two LAs due to irreconcilable boundary changes (Bournemouth, Christchurch and Poole and Dorset). The final analytic dataset covers 98.8% of the child population of England across the study period.

### Data sources and measures

Our primary outcome was the annual rate of children being placed on a CPP by the initial category of abuse ‘neglect’. Our secondary outcomes were the annual rates of (1) children being placed on a CPP by the initial categories of abuse ‘physical abuse’, ‘emotional abuse’ and ‘sexual abuse’ and (2) children starting to be looked after by the state. These data were drawn from Department for Education accredited official statistics on children in need and children looked after in England.^25 26^ Counts between one and five are suppressed in publicly available data. Suppression was rare for CPPs due to recorded neglect and emotional abuse (n=21), and common for the less frequent physical abuse (n=245) and sexual abuse CPPs (n=490) (online supplemental appendix 1). Suppressed values were therefore imputed by drawing a random integer between one and five, and robustness to imputation strategy was assessed in sensitivity analyses described below. Missing data were minimal, affecting four of 1323 LA-year observations. These observations were not available in the Department for Education data, either because they were not supplied by the LA (Norfolk 2012/2013, Hackney 2020/2021–2021/2022) or because of identified data quality issues (Hampshire 2023/2024). Analyses were conducted using all available data under a complete-case analysis approach. Complete data were available for all other variables.

Our primary exposure was an adapted version of the Small Area Mental Health Index (SAMHI), a composite annual measure of population mental health for each lower super output area (LSOA) in England, available from the Place Based Longitudinal Data Resource.^27^ The original SAMHI incorporated data on mental health-related hospital attendances, which showed major post-2019/2020 discontinuities consistent with disrupted access following pandemic restrictions (online supplemental appendix 2). Our adapted SAMHI therefore excludes this indicator, combining data on remaining indicators: antidepressant prescriptions; percentage of adult patients with new diagnoses of depression and Disability Living Allowance and Personal Independence Payments for reasons of mental ill health and learning difficulties. These indicators were rescaled to have a mean of 0 and SD of 1, and maximum likelihood factor analysis was applied to compute a single factor score based on intercorrelations among the components. We validated this approach by using it to reconstruct the SAMHI from all four original indicators and comparing it with published SAMHI data, finding close correspondence (r=0.96). We aggregated the adapted SAMHI to the upper-tier LA level, weighting by LSOA population and restandardised the resulting scores such that the mean across all LAs and years was 0 with a SD of 1. Higher scores represent worse mental health.^28^

Secondary exposures included: the annual rate of children under 16 living in relative poverty before housing costs, using the children in low-income families local area statistics^29^; and the annual employment rate for the working-age population, using Labour Force survey data.^30^ All data were either available at the upper-tier LA level, or were aggregated to this level.

Additional covariates, available for a shorter time span and used in sensitivity analyses, included: net international migration, available from the Office for National Statistics,^31^ and measures of household structure only available at regional level: the proportion of households with three or more dependent children, and the proportion of lone parent households.^32^

### Statistical analysis

**Figure 1:**
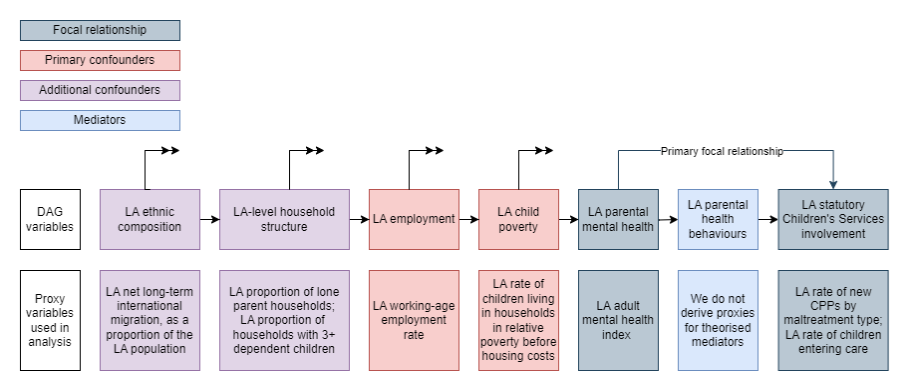
A priori directed acyclic graph (DAG). Variables with a double-headed arc are hypothesised to cause all future variables. LA, local authority; CPP, child protection plan.

We used a Directed Acyclic Graph (DAG, figure 1) to specify a priori assumptions about relationships between area-level variables, and to guide confounder selection for all analyses. It illustrates the focal relationship between adult mental ill health (the primary exposure) and statutory Children’s Services interventions (including CPPs due to recorded neglect, the primary outcome). It shows theorised confounders—including ethnic composition, household structure, employment and child poverty, ordered conceptually from more distal to more proximal determinants. And it shows theorised mediators through which the exposure may operate.

First, we computed summary statistics for all exposures, outcomes and covariates, both overall and across LAs, and visually inspected trends using summary plots at the national level and spaghetti plots at the LA level. These descriptive analyses were used to assess trends, check data integrity, identify extreme values or outliers and determine whether counts and within-LA variation were sufficient to support fixed effects estimation. This revealed the post-pandemic discontinuities in the SAMHI, leading us to compute the adapted SAMHI. We also found very small counts for CPPs due to sexual abuse, resulting in low rates with limited within-LA variation. We therefore excluded this outcome from main analyses.

Second, we fitted two-way linear fixed effects models to estimate within-LA associations between our main exposure, the adapted SAMHI and our main outcome, the rate of new CPPs due to recorded neglect, between 2014/2015 and 2022/2023. These models allow us to control for time-invariant differences between LAs and common temporal trends; we additionally controlled for child poverty and employment rates, both confounders identified in our DAG. Year fixed effects capture changes occurring across all LAs in each year, including the COVID-19 pandemic and associated disruptions to economic conditions and children’s social care processes. Since evidence suggests that child protection systems are relatively responsive to changes in socioeconomic conditions, we modelled associations within the same year.^33^ We used clustered SEs to account for within-LA serial correlation. We then repeated this process for each of our secondary outcomes, the rate of CPPs due to emotional and physical abuse, and the rate of children starting to be looked after.

Third, we fitted similar models to estimate associations of secondary exposures, child poverty and employment rates, with recorded neglect and all other maltreatment types, controlling for their respective confounders, informed by the DAG.

For all models fitted in steps 2–3, we translated estimated coefficients into counterfactual differences in the number of children affected by observed changes in exposures over the study period. We compared model-predicted outcomes under observed exposure trajectories to a counterfactual scenario in which the exposure was held constant at its 2014/2015 local-authority-specific level, leaving unaltered all other observed covariates. The 95% confidence intervals were constructed by drawing 1000 coefficient vectors from their estimated distribution, recomputing the total model-predicted additional cases for each draw, and taking the empirical 2.5th and 97.5th percentiles across the 1000 draws. The resulting estimates represent model-predicted differences in CPPs associated with observed change in exposure over the study period, relative to the counterfactual scenario. They should not be interpreted as causal estimates of attributable burden.

In sensitivity analyses, we assessed the temporal specification of the models by repeating analyses for the Neglect CPP outcome, using 1-year lagged exposures and confounders. To account for potential shared regional trends in policy and economic conditions, we re-estimated the main model additionally incorporating region fixed effects. Given sparse counts for sexual abuse, we estimated a model using a broader outcome category that combined physical and sexual abuse CPPs. To assess the robustness of our main models to residual confounding, we restricted the data to years for which additional variables household structure and migration covariates were available; these were used as proxies for the household structure and ethnic composition confounders identified in our a priori DAG. We then fitted the models outlined in steps 1–3, with and without the additional confounders. We also assessed robustness to imputation of suppressed values, fitting main models outlined in step 1, imputing extreme values (all suppressed values set to one or all values set to five). Finally, to assess the sensitivity of results to distributional and functional form assumptions, we fitted Poisson fixed effects models, using the log of the child population as the offset. All other model specifications were as in step 1.

Linear and Poisson models were estimated using the plm^34^ and fixest^35^ packages, respectively, in R V.4.1.1. Model formulae are presented in online supplemental appendix 3. Data and code are available at: https://drive.google.com/drive/folders/1VsgNEOU1diGNQVIsOAi-I4eXIrALZS2z?usp=sharing

### Patient and public involvement

The research question and primary mental health focus were defined by a care-experienced member of the research team.

Conversations with members of parent support organisations HerCircle (Newcastle)^36^ informed our contextual understanding of family experiences, and our sensitivity to the challenging distinction between intentional and unintentional harm to children.

As part of this project, we held online discussions with members of the University of Liverpool’s Young People’s Advisory Group and the Applied Research Collaboration North West Coast Public Advisor network. Participants from both groups reflected on both structural and cultural explanations for the relationship between poverty, neglect and child protection outcomes. This engagement sharpened our awareness of discursive constructions of the relationship, reinforced our analytic focus on the social determinants of family need and children’s adversity, and informed how our results are interpreted.

## Results

### Descriptive statistics

Summary statistics are presented in table 1. Across 147 LAs, between 2014/2015 and 2022/2023, rates of new CPPs were highest for recorded neglect, followed by emotional and physical abuse; recorded sexual abuse remained rare.

**Table 1:** Summary statistics for outcomes, exposures and main covariates, 147 English LAs, 2014/2015–2022/2023

Variable	Mean	SD	Min	Max	Mean within-LA SD	% variance within LA
Neglect CPP rate (per 10 000)	28.45	15	0.27	115.47	7.99	39.02
Emotional abuse CPP rate (per 10 000)	22	11.53	0.29	99.91	7.18	51.63
Physical abuse CPP rate (per 10 000)	5.16	4.61	0	39.09	2.69	49.59
Sexual abuse CPP rate (per 10 000)	2.24	1.87	0	18.66	1.33	66.13
Care entry rate (per 10 000)	29.44	11.54	6.62	94.17	5.3	26.89
Small Area Mental Health Index	0	1	−2.17	3.09	0.54	32.76
Child poverty rate (%)	19.04	7.58	4.57	43.48	1.91	7.75
Employment rate (%)	74.34	4.82	58.7	88.3	2.27	25.09

Mean within-LA SD=standard deviation over time within local authorities, averaged across all local authorities. % variance within LA=proportion of total variance attributable to within-LA variation.

CPP, child protection plan; LA, local authority.

**Figure 2:**
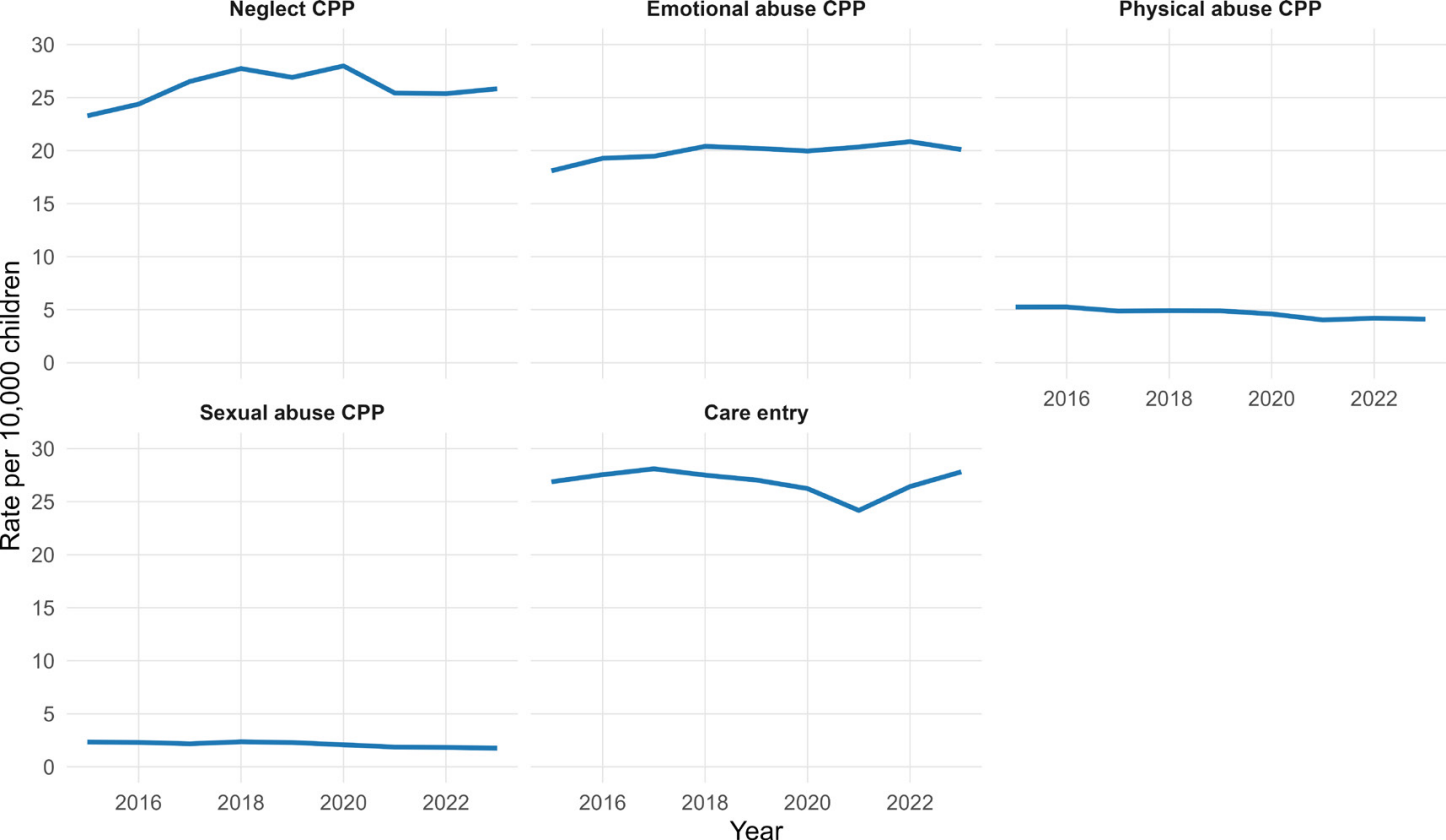
National trends in outcomes, 2014/2015–2022/2023. CPP, child protection plan.

**Figure 3:**
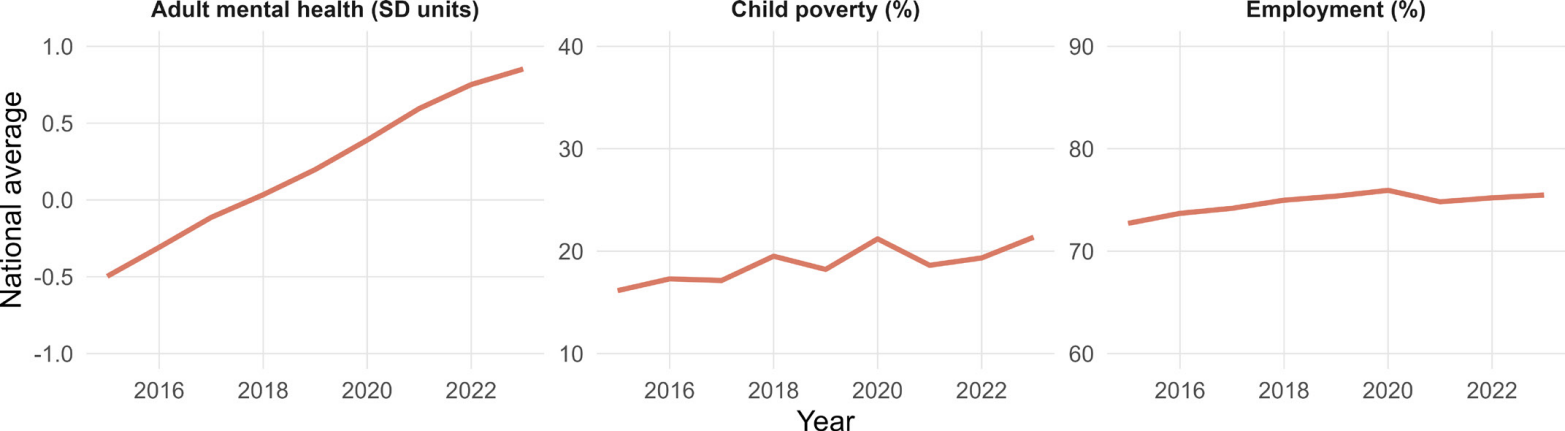
National trends in exposures, 2014/2015–2022/2023.

Figure 2 shows national trends in children’s social care involvement. Neglect CPP rates rose to 2019/2020, dipped during the pandemic and partially recovered thereafter. Care entry rates declined to 2019/2020, fell sharply in the first pandemic year and then partially recovered. Emotional, physical and sexual abuse CPP rates remained relatively stable throughout. Figure 3 shows national trends in exposures. Adult mental health steadily worsened over the study period. Child poverty increased with some fluctuations. Employment increased overall, despite a pandemic-related dip.

Spaghetti plots of LA-level trends show substantial within and between-LA variation for most outcomes, supporting our use of fixed effects models (online supplemental appendix 4). Very small absolute counts for sexual abuse CPPs, and resulting artificially high percentage variation, led us to exclude this outcome from our main analyses; results are presented in online supplemental appendix 5 for completeness.

### Model results

Table 2 summarises results from linear two-way fixed effects models for each exposure. Column 1 shows associations with SAMHI; columns 2 and 3 show associations with child poverty and employment, respectively. Table 3 presents model-based estimates of the differences in the numbers of children placed on CPPs or entering care associated with observed trends in exposures between 2014/2015 and 2022/2023, compared with a counterfactual scenario in which each exposure (SAMHI, child poverty, employment) had remained at its 2014/2015 LA-specific level, with other covariates maintained at their observed values over time.

**Table 2:** Within-local authority associations of adult mental health, child poverty and employment, with children’s social care outcomes, 2014/2015–2022/2023

Outcomes	Annual change in rate per 10 000 children (95% CI) over the study period		
	For an SD increase in the SAMHI, controlling for child poverty and employment	For a %-point increase in child poverty, controlling for employment	For a %-point increase in employment
Neglect CPP	5.28 (−0.49 to 11.04)	1.22 (0.22 to 2.23)	−0.04 (−0.34 to 0.27)
Emotional abuse CPP	3.60 (−0.83 to 8.02)	0.45 (−0.30 to 1.21)	−0.03 (−0.28 to 0.22)
Physical abuse CPP	−1.48 (−3.35 to 0.38)	0.07 (−0.15 to 0.29)	−0.10 (−0.20 to 0.00)
Care entry	4.40 (1.06 to 7.74)	0.61 (0.08 to 1.14)	−0.17 (−0.33 to −0.01)

CPP, child protection plan; SAMHI, Small Area Mental Health Index.

**Table 3:** Estimated differences in the numbers of children associated with observed trends in exposures, 2014/2015–2022/2023 relative to a counterfactual scenario of no change from baseline, trends in confounders unaltered

Outcomes	Estimated differences in the numbers of children associated with observed trends in the exposure, 2014/2015–2022/2023, trends in confounders unaltered (95% CI)		
	SAMHI	Child poverty	Employment
Neglect CPP	44 659 (−3763 to 94 911)	33 248 (5635 to 60 057)	−860 (−7381 to 5915)
Emotional abuse CPP	30 424 (−6724 to 68 860)	12 298 (−8647 to 32 401)	−651 (−5971 to 4875)
Physical abuse CPP	−12 739 (−28 411 to 3534)	1876 (−4305 to 7782)	−2178 (−4323 to 50)
Care entry	37 307 (9266 to 66 432)	16 594 (1887 to 30 784)	−3670 (−7119 to −88)

CPP, child protection plan; SAMHI, Small Area Mental Health Index.

#### Associations with SAMHI

Associations between area-level adult mental health and children’s social care involvement varied by level of involvement and maltreatment type. Between 2014/2015 and 2022/2023, within English LAs and controlling for employment and child poverty rates, higher SAMHI scores were associated with higher rates of children being placed on CPPs due to recorded neglect and emotional abuse. However, effect sizes were imprecise, with wide CIs including zero. Accordingly, rising SAMHI scores over the study period were associated with an estimated difference of 44 659 additional neglect CPPs (95% CI −3763 to 94 911) and a difference of 30 424 emotional abuse CPPs (95% CI −6724 to 68 860), relative to a counterfactual scenario in which SAMHI had remained constant from 2014/2015. There was little evidence of an association with physical abuse. Higher SAMHI was associated with higher rates of children entering care (4.40, 95% CI 1.05 to 7.74), corresponding to an estimated difference of 37 307 care entries over the study period (95% CI 9266 to 66 432).

#### Associations with child poverty

Child poverty showed a positive association with neglect CPPs. A one percentage point increase in child poverty was associated with approximately one neglect CPP per 10 000 children (1.22, 95% CI 0.22 to 2.23), equivalent to an estimated difference of 33 248 neglect CPPs overall (95% CI 5635 to 60 057). Poverty was also positively associated with care entry rates, corresponding to an estimated difference of 16 594 care entries (1887 to 30 783).

#### Associations with employment

Employment rates were not meaningfully associated with either neglect or emotional abuse CPPs. There was some evidence of an association with lower physical abuse CPP rates, but CIs included the null (−0.10, 95% CI −0.20 to 0.00). Higher employment rates were also associated with lower care entry rates (−0.17, 95% CI −0.33 to −0.01). Accordingly, overall improvements in employment over the study period were associated with an estimated difference of −2178 physical abuse CPPs (95% CI −4323 to 50), and −3670 care entries (−7119 to −88).

### Sensitivity analyses

Our sensitivity analysis using lagged exposure and confounders yielded similar findings, indicating robustness to alternative temporal specifications (online supplemental appendix 5). Results were also robust to adjustment for region-level fixed effects (online supplemental appendix 6). Results of the analysis combining sexual and physical abuse CPPs (online supplemental appendix 7) differed from those for the separately estimated physical and sexual-abuse CPP models, with negative associations observed across all exposures. Findings appear sensitive to outcome specification when abuse categories are sparse. Restricting the dataset to years 2014/2015 to 2019/20 to control for additional confounders resulted in some attenuation of the association between SAMHI and both emotional abuse CPPs (from 4.32 to 3.63) and care entry (from 4.58 to 3.81), with wide uncertainty and overlapping CIs. There was negligible change for all other exposure–outcome combinations (online supplemental appendix 8). Results were robust to imputation of minimum and maximum values (online supplemental appendix 9). Sensitivity analyses using Poisson fixed effects models showed that the variance of the outcome exceeded the conditional mean within LAs, and cluster-robust SEs were used to account for clustering at the LA level in the estimation of CIs. Incidence rate ratios were generally consistent in direction with the linear fixed effects results, although CIs more frequently included the null. Results were most robust for SAMHI and care entry (online supplemental appendix 10).

## Discussion

### Summary

In this longitudinal ecological analysis of English LAs (2014/2015–2022/2023), worsening adult mental health was associated with higher rates of children being placed on CPPs due to recorded neglect and emotional abuse, but not physical abuse, after controlling for key confounders—but substantial uncertainty limits strong inference. Among area-level social and economic factors, child poverty showed an association with higher rates of CPPs due to recorded neglect, with little evidence of comparable associations for recorded emotional and physical abuse. There was weak evidence that higher employment rates were associated with lower rates of CPPs due to recorded physical abuse only, again with wide uncertainty. Care entry, the most acute children’s social care outcome, was associated with all three exposures: rates increased with worsening adult mental ill health and higher child poverty, and decreased with higher employment.

Together, these findings suggest that the occurrence and identification of neglect may reflect broader social conditions and stressors potentially affecting parental caregiving capacity and service response.^37 38^ At the level of underlying need, socioeconomic conditions and material stress may affect parents’ ability to cope and draw the notice of child protection systems.^39^ Negative perceptions of parents in poverty^39^ and conditions of resource constraint may shape service delivery, affecting quality, workforce capacity and thresholds for intervention.^40–42^

Our results broadly align with the wider literature, with some divergences. Our finding of potential positive associations of mental ill health with both recorded neglect and emotional abuse is consistent with systematic review evidence identifying parental mental ill health as a shared risk factor across abuse types—though our null finding for recorded physical abuse diverges from these reviews.^14 16^ In our analyses, associations appeared larger for recorded neglect than for emotional abuse—although with wide CIs—whereas pooled ORs from Luo *et al*’s meta-analysis were of broadly similar magnitude across neglect, emotional and physical abuse.

Similarly, our findings of a positive association between child poverty and recorded neglect align with systematic review evidence—but we find no clear associations with recorded emotional and physical abuse where reviews report positive associations of comparable magnitude.^14 16^ Nevertheless, across the literature as a whole, poverty is more consistently associated with neglect than other maltreatment types,^43^ and a neglect-specific finding is shown in some individual^19^ and neighbourhood-level longitudinal studies.^19 44^ Our estimated association of child poverty with care entry is consistent with our 2020 analysis using similar data and methods^24^ but a more recent study found no evidence of an association between within-area child poverty and care entry. Differences in outcome definition, temporal adjustment, and statistical model specification may contribute to differences in findings.^45^

Our findings for employment are also consistent with the systematic review evidence, though the evidence base shows heterogeneity. The association with physical abuse is common,^14 46 47^ but one recent study found an association with neglect only, raising the possibility of more complex or nuanced pathways linking employment and child maltreatment.^48^ Apparently discordant findings may also reflect differential effects of employment by parents’ gender. One study found that child maltreatment risk was negatively associated with male employment and positively associated with female employment, suggesting that risk may reflect gender differences in caregiving roles and the ways in which economic shocks influence family time and family stress.^17 18 33^

Overall, the mix of concordant and divergent findings is expected. Heterogeneity in the definition and measurement of exposures and outcomes, as well as in model specification, is likely to yield varied results. Relationships between social and health determinants and children’s social care involvement are also likely to be shaped by complex socio-cultural contexts, policy environments, shock events and child protection systems.^49^

### Limitations

This study has important limitations. First, the ecological design means that findings reflect results at the LA level, not the individual level. They cannot determine whether families of children being placed on a CPP are also experiencing worsening adult mental health, poverty or employment changes occurring within their LA. At the LA level, the processes shaping exposures and covariates are less readily disentangled, contributing to wider uncertainty in adjusted estimates; exposures should be interpreted as contextual indicators of area-level mental health, economic conditions and labour market participation, rather than direct measures of parental mental health, family poverty or family employment. Moreover, our Children’s Services outcomes reflect both underlying need and service response, further limiting our understanding of the mechanisms linking exposures and outcomes. Nevertheless, the LA is a policy-relevant unit of analysis, and our study captures complete data for a critical period in England, spanning post-recession austerity years, pandemic disruption and a period of rising living costs. Further research should investigate mechanisms at the child and family level in England, and undertake mediation analyses to identify priority targets for intervention.

Second, our main exposure, the adapted SAMHI, may be a crude proxy for area-level parental mental health, the phenomenon of interest. The SAMHI is composed of indicators that reflect mental health service access or treatment, and may not be fully representative of underlying area-level mental health. Further research could incorporate self-reported measures of psychological distress, which are less dependent on service access.^28^ Similarly, outcome measurement is child protection systems-driven, and child maltreatment often goes unreported.^1^ Greater surveillance of families seeking mental health support or experiencing disadvantaged social conditions may drive systems patterns.^43^

Third, neighbouring LAs may share evolving policy environments, economic conditions and service structures. Although our sensitivity analysis using both LA and region fixed effects suggests that this is unlikely to materially affect our results, residual spatial dependence in time-varying unobserved factors may remain.

Fourth, sensitivity analyses adjusting for additional confounders showed minimal attenuation of estimates, but some of the confounders were only available at regional level, or were imperfect proxies for DAG variables. Residual confounding due to these and other time-varying factors (eg, housing insecurity, school closures), is possible.

Finally, though this study makes a key contribution, showing the contribution of contemporary trends in area-level mental health and economic conditions to trends in costly child protection and care interventions, these associations are well established in the wider international evidence base. To galvanise new policy efforts, robust evaluations of major policies and interventions are needed.

### Implications for policy

Our findings have a number of implications for population-level policy. Together with the broader evidence base on parental mental ill health as a common and salient factor in children’s social care involvement,^50 51^ they suggest that supporting adult mental health is not only a health priority, but also a child protection imperative. Timely, accessible mental health support for parents, alongside cross-sector collaboration across health and social care, adult and children’s services, may help address conditions associated with identified neglect, emotional abuse and care entry.

The suggestive association between child poverty and recorded neglect, and the association with care entry in the main model, lend additional weight to calls for national anti-poverty and poverty-reduction strategies. There is increasing evidence that policies that increase household income and buffer against economic shocks can help alleviate the social conditions that drive neglect and poor subsequent adolescent health outcomes.^37 52 53^ For national policymakers, a first step might be setting child poverty targets.^54^ At a local level, practitioners should be supported to recognise child poverty as a modifiable factor associated with neglect and care entry,^10 18^ and address it where possible.^55^ Given the conceptual and operational challenges in defining and responding to neglect, greater consistency in recording families’ specific socioeconomic circumstances and health needs may help direct appropriate support to those experiencing adversity associated with neglect.^51^

The findings resonate with recent legal developments in some US jurisdictions. In California, for example, Assembly Bill 2085 codifies the distinction between material hardship and child neglect.^56^ Updated guidance to child welfare agencies insists on the distinction in practice, encourages referral to supportive services for families with economic needs, and advises aligning local policies with the statutory requirements.^57^ This does not resolve a fundamental tension—that poverty can create the conditions that undermine safe caregiving and cause harm.^58^ But legal frameworks and reforms can help shift culture and practice.^50^

In England, successive policies and practice models have aimed to promote less adversarial approaches to family support, including greater attention to parental mental health and wider family needs, alongside child protection activity.^59–61^ However, strengthening statutory Section 17 support, which places a duty on LAs to safeguard and promote the welfare of children in need, including through financial and practical assistance to families, could help further mitigate the risk of recorded neglect.^62^

Finally, policymakers should consider our findings of a modest negative association of employment with care entry, alongside broader uncertain evidence of the effect of maternal employment. Overall, policies that promote secure, adequately paid and flexible work, can help parents provide for their children and spend time with their children.

## Conclusions

In summary, this study highlights that area-level adult mental health, child poverty and employment are important upstream determinants of children’s social care involvement. Recorded neglect and care entry appear particularly sensitive to broader social and economic stressors; emotional and physical abuse show more mixed patterns. These findings underscore that often, “children in need have parents in need” (p.22).^63^ Interventions must extend beyond traditional child protection measures to address overarching social conditions. Supporting adult mental health, implementing effective poverty-reduction strategies and supporting family-friendly work policies can help mitigate the risk of neglect and reduce care entry. Legal and statutory frameworks can help reinforce a strong culture of ordinary help for families experiencing financial stress.^64^ Together, these findings emphasise the importance of integrated, preventive approaches that address the social contexts shaping social care involvement.

## Supplementary material

10.1136/bmjopen-2026-119211 online supplemental file 1

## Data Availability

All data relevant to the study are included in the article or uploaded as supplementary information.
