# Supplementary material for: Is neglect different? Social determinants of child protection plans by recorded maltreatment type in England: a longitudinal ecological study, 2014/2015–2022/2023
Source: BMJ Open. 2026 Aug 5;16(8):e119211. doi: 10.1136/bmjopen-2026-119211 (PMC13448618; doi:10.1136/bmjopen-2026-119211)
Supplement: Supplementary file 1 [file bmjopen-2026-119211.reviewer_comments.pdf]

PEER REVIEW HISTORY

BMJ Open publishes all reviews undertaken for accepted manuscripts. Reviewers are asked to complete a checklist review form and are provided with free text boxes to elaborate on their assessment. These free text comments are reproduced below.

ARTICLE DETAILS

Title (Provisional)

Is neglect different? Social determinants of child protection plans by recorded maltreatment type in England: a longitudinal ecological study, 2014/15-2022/23

Authors

Murley, Francesca (Joint first author); Gecer, Nurgul (Joint first author); Niccodemi, Gianmaria; Black, Michelle; Fahy, Katie; Taylor-Robinson, David; McHale, Philip; Bennett, Davara

---

VERSION 1 - REVIEW

**Reviewer**	**1**
**Name**	**Kelson, Zoe**
**Affiliation**	**University of Exeter, Mathematics**
**Date**	**25-Mar-2026**
**COI**	**None**

This longitudinal ecological study aimed to assess the associations of area-level adult mental health, child poverty, and employment, with recorded neglect, and to compare these associations with those observed for other forms of maltreatment.

Reviewer Comments:

"used two-way linear fixed effects models to estimate associations between each exposure and outcome, controlling for confounders" [Abstract]

The statistical methods are summarised in the Abstract by the authors.

"Rising adult mental ill health was associated with substantially higher rates of neglect and emotional abuse CPPs, though confidence intervals spanned the null...

Higher employment was associated with a small reduction in physical abuse CPPs, with confidence intervals including the null"

" [Abstract]

Can the authors please revise inferences of association (i.e. 'substantially higher rates' or a 'small reduction') given the statistically non-significant outcomes?

"147 upper-tier local authorities in England from 2014/15 to 2022/23...

Children aged 0-17 years" [Abstract]

and

"We undertook a longitudinal, ecological study at the upper-tier local authority level in England, using panel data from 147 local authorities between 2014/15 and 2022/23. During this time, some local authorities underwent boundary changes, including splits and mergers. Where possible, we harmonised these data, retaining the larger boundary. We excluded two local authorities due to their very small population size (City of London and Isles of Scilly), and two local authorities due to irreconcilable boundary changes (Bournemouth, Christchurch and Poole, and Dorset)."

Can the authors please demonstrate whether the included data can be considered to be representative?

Can the authors please tabulate cohort demographics and characteristics?

"Counts between one and five are suppressed in publicly available data. Suppressed values were therefore imputed by drawing a random integer between one and five."

The authors suitably conduct a sensitivity analysis on the approach taken for imputing suppressed data.

"Data for only four local authority-years were not available (Norfolk 2012/13, Hackney 2020/21–2021/22, Hampshire 2023/24). They were retained as missing due to the minimal extent of missingness."

Can the authors please assess whether missing data can be considered to be missing at random?

Can the authors please clarify how this missing data was handled in the analysis?

"Our primary exposure was the Small Area Mental Health Index (SAMHI), a composite annual measure of population mental health for each lower super output area (LSOA) in England, available from the Place Based Longitudinal Data Resource, and adapted to take into account COVID related discontinuities (Appendix 1).23 The adapted SAMHI combines data on: antidepressant prescriptions; percentage of adult patients with new diagnoses of depression; and Disability Living Allowance and Personal Independence Payments for reasons of mental ill health and learning difficulties. We aggregated the adapted SAMHI to the upper-tier local authority level, weighting by LSOA population, and re-standardised the resulting scores such that the mean across all local authorities and years was 0 with a standard deviation of 1. "

Can more detail please be provided in the main article on how this index has been adapted, aggregated, weighted, and re-standardised?

"Additional covariates, available for a shorter time span and used in sensitivity analyses, included: net international migration, available from the Office for National Statistics,30 and measures of household structure only available at Regional level: the proportion of households with three or more dependent children, and the proportion of lone parent households."

Can the authors please confirm if there was any missing covariate data?

Can the authors please clarify the inclusion of ethnicity data in the model, and specify where this was sourced from?

"We also found very small counts for CPPs due to sexual abuse, resulting in low rates with limited within-local authority variation. We therefore excluded this outcome from main analyses"

Can this outcome (sexual abuse) please be combined with a different outcome (e.g. physical abuse) in a sensitivity analysis?

"we fitted two-way linear fixed effects models to estimate within-local authority associations between our main exposure, the adapted SAMHI, and our main outcome, the rate of new CPPs due to neglect, between 2014/15 and 2022/23. These models allow us to control for time-invariant differences between local authorities and common temporal trends; we additionally controlled for confounders identified in our Directed Acyclic Graph (DAG, Figure 1) - child poverty and employment rates. We used clustered standard errors to account for within-local authority serial correlation. We then repeated this process for each of our secondary outcomes, the rate of CPPs due to emotional and physical abuse, and the rate of children starting to be looked after. Third, we fitted similar models to estimate associations of secondary exposures, child poverty and employment rates, with neglect and all other maltreatment types, controlling for their respective confounders based on our DAG (Figure 1)"

An appropriate modelling methodology has been undertaken and is clearly described by the authors.

Did the authors consider performing joinpoint regression analysis to analyse changes in trends over time?

"Uncertainty in counterfactual estimates was quantified via Monte Carlo simulation, drawing 1000 coefficient vectors from the multivariate normal distribution, with mean equal to the regression-estimated coefficients and covariance equal to the regression-estimated variance-covariance matrix. "

The authors do well to quantify and communicate uncertainty.

"In sensitivity analyses, to assess the robustness of our main models to residual confounding, we restricted the data to years for which additional household structure and migration confounders were available. We then fitted the models outlined in steps 1-3, with

and without the additional confounders. We also assessed robustness to imputation of suppressed values, fitting main models outlined in step 1, imputing extreme values (all suppressed values set to one or all values set to five). Finally, to assess the sensitivity of results to distributional and functional form assumptions, we fitted Poisson fixed effects models, using the log of the child population as the offset. All other model specifications followed those outlined in step 1. "

A number of valuable sensitivity analyses have been explored by the authors.

"Between 2014/15 and 2022/23, within English local authorities and controlling for employment and child poverty rates, higher SAMHI scores were associated with higher rates of children being placed on CPPs due to neglect and emotional abuse. Estimated effect sizes were substantial but imprecise, with wide confidence intervals including zero. Rising SAMHI scores over the study period were associated with an estimated 44,659 additional neglect CPPs (95% CI -3,763 to 94,911), and 30,424 additional emotional abuse CPPs (95% CI -6,724 to 68,860), relative to a counterfactual scenario in which SAMHI had remained constant from 2014/15"

and

"There was some evidence of an association with lower physical abuse CPP rates, although the confidence intervals included the null (-0.10, 95% CI -0.20 to 0.00). Higher employment rates were also associated with lower care entry rates (-0.17, 95% CI -0.33 to -0.01). Overall improvements in employment over the study period were associated with an estimated 2,178 fewer physical abuse CPPs (95% CI -4,323 to 50), and 3,670 fewer care entries (-7,119 to -88). "

Can the authors please revise inferences of association throughout the Results given the statistically non-significant outcomes?

Figures: Can confidence intervals please be plotted on all figures?

Limitations - Use of population-level administrative data reflecting both underlying need and service response - Ecological design limits inference to area-level associations" [Strengths and Limitations of this Study]

and

"This study has important limitations. First, the ecological design means that findings reflect results at the local authority level, not the individual level. The local authority is a policyrelevant unit of analysis, and our study captures complete data for a critical period in England, spanning post-recession austerity years, pandemic disruption, and a period of rising living costs. Further research should investigate mechanisms at the child and family level in England, and undertake mediation analyses to identify priority targets for intervention. Second, our main exposure, the adapted SAMHI, may be a crude proxy for parental mental health, the phenomenon of interest. The SAMHI is composed of indicators that reflect mental health service access or treatment, and may not be fully representative of underlying

mental health. Further research could incorporate self-reported measures of psychological distress, which are less dependent on service access.27 Similarly, outcome measurement is child protection systems-driven, and child maltreatment often goes unreported. 1 Greater surveillance of families seeking mental health support or experience disadvantaged social conditions may drive systems patterns.37 Third, sensitivity analyses adjusting for additional confounders showed minimal attenuation of estimates, but some of confounders were only available at regional level and residual confounding is possible. Finally, though this study makes a key contribution, showing the contribution of contemporary trends in mental health and economic conditions to trends in costly child protection and care interventions, these associations are well established in the wider international evidence base. To galvanise new policy efforts, robust evaluations of major policies and interventions are needed. "

A Discussion on the study limitations is provided by the authors.

Thanks for providing a copy of the STROBE checklist.

**Reviewer**	**2**
**Name**	**Hare, Megan**
**Affiliation**	**Tulane University School of Medicine**
**Date**	**07-May-2026**
**COI**	**None**

The longitudinal ecological design, use of national administrative data across English local authorities, and comparison across maltreatment categories are notable strengths. At the same time, there are several conceptual, analytic, and interpretive issues that should be addressed to strengthen the rigor and clarity of the paper. In particular, the manuscript would benefit from more cautious interpretation of ecological associations, clearer handling of uncertainty surrounding several key estimates, and additional discussion of how administrative child protection data reflect both underlying need and service response processes. Some claims regarding neglect-specific associations also appear stronger than the presented evidence fully supports. Addressing these issues would substantially improve the manuscript's interpretability and contribution.

1. The opening paragraph establishes the importance of neglect and the policy relevance of reducing statutory child protection involvement, but the flow of the introduction feels somewhat choppy. The manuscript moves quickly from prevalence and policy priorities to definitions of neglect, conceptual debates, social workers' perceptions, and then empirical antecedents of maltreatment. As written, the connection among these ideas is not always clear. For example, the sentence describing social workers' recognition of parental mental health and addiction, but limited recognition of poverty, feels somewhat abrupt in its current placement. Similarly, the transition to "These conceptual and practical challenges

have prompted empirical research into the antecedents of different types of child maltreatment" is not fully developed. The authors should revise the introduction to more clearly build the logic of the study: why neglect may differ conceptually from other maltreatment types, why social determinants such as poverty and adult mental health may be especially relevant to neglect, and why comparing neglect with other maltreatment categories is necessary.

2. The PPI section is a strength and provides valuable context for how lived experience informed the framing and interpretation of the study. However, the current wording feels somewhat informal and includes personal biographical details about one author and their family that may be unnecessary or potentially identifying in a peer-reviewed manuscript. The sentence about including a future digital story also reads as provisional and may not be appropriate for the final manuscript. I recommend revising this section to focus more broadly on how lived experience and public advisory input shaped the research question, interpretation, and dissemination.

3. Although the authors use a longitudinal fixed-effects design, the study remains ecological and observational. Some wording implies stronger causal inference than the design can support, particularly language around "drivers," "determinants," and the estimated number of additional cases associated with exposure trends. The authors should more consistently frame findings as area-level associations and clarify that the counterfactual estimates are model-based projections rather than causal estimates of attributable burden.

4. The manuscript frames child poverty as having a specific association with neglect compared with other maltreatment types. However, it is not clear that the authors formally tested whether coefficients differed across maltreatment categories. Given overlapping confidence intervals across outcomes, the evidence for specificity should be interpreted cautiously. The authors should either formally test differences across outcomes or soften this language.

5. Several central findings, particularly those involving SAMHI and neglect or emotional abuse CPPs, have wide confidence intervals that include the null. Although the estimated effect sizes are meaningful, the uncertainty is substantial. The Discussion and Abstract should more clearly distinguish between robust findings and suggestive patterns. Relatedly, the abstract states that adult mental ill health was associated with substantially higher rates of neglect and emotional abuse CPPs, although confidence intervals spanned the null. This should be worded more cautiously so the abstract does not overstate the strength of evidence.

6. Child Protection Plans reflect both underlying maltreatment risk and the functioning of the child protection system. This is especially important for neglect, which is more dependent on professional judgment, local thresholds, socioeconomic surveillance, and service availability than some other maltreatment categories. The manuscript would benefit from deeper consideration of whether observed associations reflect changes in neglect

itself, changes in identification, or changes in administrative responses. This discussion should also consider whether changes in local thresholds, workforce capacity, funding cuts, inspection pressures, policy reforms, social care staffing shortages, or referral pathways may have influenced CPP rates independently of actual maltreatment need.

7. The use of linear fixed-effects models is reasonable and clearly presented, but the outcomes are rates derived from count data. The Poisson sensitivity analyses showed substantial overdispersion, which raises questions about the best modeling approach. The authors should provide a clearer justification for using linear fixed-effects models as the primary analytic strategy and explain whether alternative count models, such as negative binomial models, were considered.

8. The estimated numbers of additional CPPs or care entries are useful for policy communication, but they depend heavily on the model specification and assumptions. The manuscript should be clearer that these estimates should not be interpreted as the number of cases that would necessarily have been prevented if poverty or adult mental ill health had remained unchanged.

9. The manuscript would benefit from additional justification of the temporal structure of the models. Exposures and outcomes appear to be modeled within the same fiscal year, but it is plausible that changes in poverty, employment, or adult mental health may influence child protection outcomes with temporal lag. Some effects may emerge after months or years. Discussion of why contemporaneous associations were selected, and whether lagged models were explored, would strengthen the analytic rationale.

10. Adult mental health, child poverty, and employment are conceptually and statistically related constructs. The manuscript would benefit from additional discussion of potential multicollinearity among exposures and how this may influence coefficient stability and interpretation within the fixed-effects models.

11. The analyses assume independence across local authorities, yet neighboring local authorities may share policy environments, economic conditions, or regional service structures. The manuscript would benefit from discussion of potential spatial dependence and whether spatial clustering or regional autocorrelation may influence estimates.

12. Additional detail regarding the frequency and distribution of suppressed/imputed values across outcomes and years would improve transparency. Because low-count suppression may disproportionately affect certain maltreatment categories, especially sexual abuse CPPs, the potential impact of this imputation strategy on estimates should be discussed more explicitly. Although the random imputation of suppressed counts between one and five seems reasonable and sensitivity analyses are reassuring, the manuscript should more explicitly state how many values were suppressed by outcome type.

13. The area-level exposure measures may not capture the family-level mechanisms implied in parts of the interpretation. SAMHI captures area-level adult mental health, not specifically

caregiver or parental mental health; employment is working-age population employment, not parent employment; and poverty is measured at the area level rather than the family level. The manuscript should more clearly acknowledge that these measures may function as broad area-level indicators rather than direct measures of caregiving capacity, caregiver employment conditions, or family economic hardship.

14. The appendix provides helpful detail, but because the adapted SAMHI is central to the analysis, the main text should briefly explain why the original index was modified and what indicator was removed.

15. The manuscript should specify whether SAMHI was standardized across all local authorities and years combined or within each year, as this affects interpretation of a one-standard-deviation increase.

16. Table 3 notes that highlighted cells denote associations of interest based on effect size and confidence intervals, but no formal cutoff was used. This could unintentionally guide readers toward selected interpretations. Consider removing the highlighting or defining objective criteria.

17. The manuscript should consistently distinguish among “neglect,” “recorded neglect,” and “CPPs due to neglect.” This distinction is important because the study measures administrative child protection outcomes, not maltreatment prevalence directly.

18. The study period spans COVID-19 disruptions, which likely affected both exposure measurement and child protection processes. The manuscript would benefit from a clearer explanation of how year fixed effects and the adapted SAMHI address these disruptions.

19. The Methods mention that boundary changes were harmonized where possible, but additional detail would improve reproducibility.

20. The policy section is compelling, but some recommendations imply that the observed associations directly identify intervention targets. The authors should ensure that policy implications remain proportionate to the ecological evidence.

21. The heading appears to state “2014/15-20219/20,” which should likely be corrected to “2014/15-2019/20.”

22. The manuscript states that all data are aggregate and publicly available, and that code is available from the corresponding author on request. Given BMJ Open’s emphasis on transparency, the authors should consider providing direct links to the datasets and making analytic code available in a public repository or explaining why this is not possible.

23. The authors state that confounders were identified using a DAG, but the rationale for included and excluded variables is not fully explained. A clearer explanation of the assumed relationships among SAMHI, poverty, employment, and CPP outcomes would help readers evaluate the adjustment strategy.

24. The manuscript notes the ecological design, but it could more explicitly state that the analysis cannot determine whether children entering CPPs are in the same families or areas experiencing worsening adult mental health, poverty, or employment changes. This limits conclusions about mechanisms linking social determinants to neglect.

25. Fixed effects handle stable differences across local authorities, but not changing time-varying contextual factors such as local service funding, housing insecurity, migration, school closures, social care staffing shortages, or changes in referral practices. These sources of residual time-varying confounding should be acknowledged more explicitly.

VERSION 1 - AUTHOR RESPONSE

Response to Reviewers' comments

**Reviewer #1**<br>Comments are numbered, for ease of reference.	**Authors' response**	**Corrections**<br>Page and line numbers cited in this section correspond to the simple revised manuscript, in which tracked changes have been accepted. In this column, for the sake of clarity, new material is underlined.
1. Can the authors please revise inferences of association (i.e. 'substantially higher rates' or a 'small reduction') given the statistically non-significant outcomes?	Thank you for this. We have now revised these inferences to more appropriately convey the uncertainty. We still report on the direction of associations as the data are for the whole population rather than derived from a sample.	Page 3, lines 77-79<br><br>Rising adult mental ill health was associated with higher rates of neglect and emotional abuse CPPs at area-level, but confidence intervals included the null.<br><br>Page 3, lines 81-82<br><br>Higher employment was associated with fewer physical abuse CPPs, though confidence intervals included the null.
2. Can the authors please demonstrate whether the included data can be considered to be representative?	Thank you for this question. The study offers near-complete coverage of upper-tier local authorities in England. Two outlier local authorities (City of London and Isles of Scilly) were excluded due to very small population sizes, and two (Dorset, and Bournemouth, Christchurch and Poole) due to boundary changes that prevented consistent harmonisation across the study period.<br><br>These exclusions are minimal relative to the total number of upper-tier local authorities, and are consistent with approaches taken in other ecological, area-level studies in England. See for example:<br>Alexiou A, Fahy K, Mason K, *et al.* Local government funding and life expectancy in England: a longitudinal ecological study. *The Lancet Public Health* 2021; **6**: e641–7. http://www.thelancet.com/article/S2468266721001109/fulltext<br><br>Overall, the final dataset covers 98.8% of the child population of England across the study period, effectively indicating complete coverage. We have now clarified this in the Methods.	Page 5, lines 181-182<br><br>The final analytic dataset covers 98.8% of the child population of England across the study period.

3. Can the authors please tabulate cohort demographics and characteristics?	Many thanks. Since this is an ecological longitudinal study using local authority-level panel data, there is no individual-level cohort from which demographic characteristics can be drawn. We have instead provided descriptive statistics for all included variables at the local authority level, including measures of within- and between- area variation (Table 1). Our aim was to provide a full and appropriate description of the analytic sample for this study design.	NA
4. Can the authors please assess whether missing data can be considered to be missing at random?	Thank you for this question. Missing data in the analytic dataset are extremely limited, affecting only four of 1,323 local authority-year observations in the Children In Need Census.<br><br>The missing data are for Norfolk in 2013, Hackney council in 2021 and 2022, and Hampshire in 2024. There is no indication that missingness is related to outcome levels. Issues relating to Hackney and Hampshire are outlined in the methodology document here and excerpted below: https://explore-education-statistics.service.gov.uk/methodology/children-in-need<br><br>"**Hackney, 2022, 2021**<br>Hackney had a cyberattack in December 2020, which had a significant impact on their information management systems in subsequent years."<br><br>"**Hampshire, 2024**<br>Hampshire local authority moved to a new case management and reporting system and their return in 2024 had significant data quality issues and was assessed to not be sufficiently reliable to use."<br><br>There is less detailed information on Norfolk in 2013, as is common with older publicly available data. The methodology document states:<br><br>**Norfolk, 2013**<br>"Norfolk local authority did not provide a CIN return within the required timescales."<br>https://assets.publishing.service.gov.uk/media/5a75a530e5274a4368298dca/SFR45-2013_Methodology_Document.pdf<br><br>Given the small amount of missing data, we judged that formal modelling of missing data would not add value. Analyses therefore used all available data under a complete-case approach within the fixed effects modelling framework. We have now clarified our approach in the manuscript.	Page 6, lines 193-198<br><br>Missing data were minimal, affecting four of 1,323 local authority-year observations. These observations were not available in the Department for Education data, either because they were not supplied by the local authority (Norfolk 2012/13, Hackney 2020/21–2021/22) or because of identified data quality issues (Hampshire 2023/24). Analyses were conducted using all available data under a complete-case analysis approach. Complete data were available for all other variables.
5. Can the authors please clarify how this missing data was handled in the analysis?	Thank you – analyses were conducted on the resulting unbalanced panel using a complete-case approach: all local authority-years with complete data on all variables were included in the two-way fixed effects model. We have now stated this more explicitly in the manuscript.	Page 6, lines 197-198<br><br>Analyses were conducted using all available data under a complete-case analysis approach.
6. Can more detail please be provided in the main article on how this index has been adapted, aggregated,	Thank you for this and we agree that this would improve the clarity – we have shifted some of the detailed previously covered in the appendix into the main body of the manuscript.	Page 6, lines 199-214<br><br>Our primary exposure was an adapted version of the Small Area Mental Health Index (SAMHI), a composite annual measure of population mental health for each lower super output area (LSOA) in

weighted, and re-standardised?		England, available from the Place Based Longitudinal Data Resource.[27] The original SAMHI incorporated data on mental health-related hospital attendances, which showed major post-2019/20 discontinuities consistent with disrupted access following pandemic restrictions (Appendix 2). Our adapted SAMHI therefore excludes this indicator, combining data on remaining indicators: antidepressant prescriptions; percentage of adult patients with new diagnoses of depression; and Disability Living Allowance and Personal Independence Payments for reasons of mental ill health and learning difficulties. These indicators were rescaled to have a mean of 0 and standard deviation of 1, and maximum likelihood factor analysis was applied to compute a single factor score based on intercorrelations among the components. We validated this approach by using it to reconstruct the SAMHI from all four original indicators and comparing it with published SAMHI data, finding close correspondence (r=0.96). We aggregated the adapted SAMHI to the upper-tier local authority level, weighting by LSOA population, and re-standardised the resulting scores such that the mean across all local authorities and years was 0 with a standard deviation of 1. Higher scores represent worse mental health.[28]
7. Can the authors please confirm if there was any missing covariate data?	Yes, thank you – we confirm that there were no other missing data, and have clarified in the manuscript.	Page 6, line 198<br><br>Complete data were available for all other variables.
8. Can the authors please clarify the inclusion of ethnicity data in the model, and specify where this was sourced from?	Thank you for this comment. We did not directly include ethnicity in our regression models. However, since we had identified it as a potentially influential confounder in our a priori Directed Acyclic Graph, we used a partial proxy that was available, LA net long-term international migration. We have now clarified this in the methods section.	Page 7, lines 272-276<br><br>"To assess the robustness of our main models to residual confounding, we restricted the data to years for which additional variables household structure and migration covariates were available; these were used as proxies for the household structure and ethnic composition confounders identified in our a priori DAG."
9. Can this outcome (sexual abuse) please be combined with a different outcome (e.g. physical abuse) in a sensitivity analysis?	Thank you for this. We have now performed this sensitivity analysis to assess whether findings are sensitive to the specification of a relatively sparse outcome category. Results are reported in appendix 4. We have amended to manuscript accordingly..	Page 7, lines 270-272<br><br>Given sparse counts for sexual abuse, we estimated a model using a broader outcome category than combined physical and sexual abuse CPPs.<br><br>Page 11, lines 367-371<br><br>Results of the analysis combining sexual and physical abuse CPPs (Appendix 7)

		differed from those for the separately estimated physical and sexual-abuse CPP models, with negative associations observed across all exposures. Findings appear sensitive to outcome specification when abuse categories are sparse.<br><br>Supplementary materials, page 6, lines 117-125<br><br>**Appendix 7. Linear fixed effects models for CPPs due to recorded sexual abuse (low count outcome), and CPPs due to both recorded physical and sexual abuse (combined outcome)**<br>The sexual abuse CPP outcome was excluded from main analyses due to very low counts, high levels of data suppression, and limited within–local authority variation. Results are presented in Table 4 for completeness. In a sensitivity analysis, we combined sexual and physical abuse CPPs.
10. Did the authors consider performing joinpoint regression analysis to analyse changes in trends over time?	Thank you for this. We did not undertake joinpoint regression in this paper, as our main aim was not to formally model changes in trends over time. The descriptive figures are presented for context and to illustrate national-level trends in the whole population. Our inferential analysis is based on the two-way fixed effects models, which account for common time trends across local authorities via year effects.	NA
11. Can the authors please revise inferences of association throughout the Results given the statistically non-significant outcomes?	Thank you, we have now made a number of revisions, including greater caution in descriptions of the magnitude of associations where there is high uncertainty, and using more careful language when contextualising effect size to avoid implying causality.	Page 9, lines 326-330<br><br>Table 3 presents model-based estimates of the differences in the numbers of children placed on CPPs or entering care associated with observed trends in exposures between 2014/15 and 2022/23, compared to a counterfactual scenario in which each exposure (SAMHI, child poverty, employment) had remained at its 2014/15 LA-specific level, with other covariates maintained at their observed values over time.<br><br>Page 10, lines 335-336<br><br>Estimated differences in the numbers of children associated with observed trends in exposures<br><br>Page 10, lines 342-349<br><br>However, effect sizes were imprecise, with wide confidence intervals including zero. Accordingly, rising SAMHI scores over the study period were associated with an estimated difference of 44,659 additional neglect CPPs (95% CI -3,763 to 94,911),

		and a difference of 30,424 emotional abuse CPPs (95% CI -6,724 to 68,860), relative to a counterfactual scenario in which SAMHI had remained constant from 2014/15. There was little evidence of an association with physical abuse. Higher SAMHI was associated with higher rates of children entering care (4.40, 95% CI 1.05 to 7.74), corresponding to an estimated difference of 37,307 care entries over the study period (95% CI 9,266 to 66,432).<br>Page 10, lines 350-355<br><br>Child poverty showed a positive association with neglect CPPs. A one percentage point increase in child poverty was associated with approximately one neglect CPP per 10,000 children (1.22, 95% CI 0.22 to 2.23), equivalent to an estimated difference of 33,248 neglect CPPs overall (95% CI 5,635 to 60,057). Poverty was also positively associated with care entry rates, corresponding to an estimated difference of 16,594 care entries (1,887 to 30,783).<br><br>Pages 10-11, lines 356-362<br><br>Employment rates were not meaningfully associated with either neglect or emotional abuse CPPs. There was some evidence of an association with lower physical abuse CPP rates, but confidence intervals included the null (-0.10, 95% CI -0.20 to 0.00). Higher employment rates were also associated with lower care entry rates, with wide uncertainty (-0.17, 95% CI -0.33 to -0.01). Accordingly, overall improvements in employment over the study period were associated with an estimated difference of -2,178 physical abuse CPPs (95% CI -4,323 to 50), and -3,670 care entries (-7,119 to -88).
12. Can confidence intervals please be plotted on all figures?	Thank you for this. We have not included confidence intervals because the trends are based on near-complete national administrative records rather than samples and therefore do not reflect sampling variation. The Figures represent rates for the whole population, and are intended to provide descriptive trends. Statistical uncertainty is instead addressed in our regression models, which provide the main inferential framework for the study.<br><br>We recognise that interest in confidence intervals may also reflect questions about uncertainty related to data construction and aggregation. For all outcomes, counts were aggregated up to the national level before deriving rates. The same approach was used for exposures child poverty and employment rates.<br><br>Although employment data are derived from the Annual Population Survey and rates are accompanied by confidence	NA

	intervals in source data, these intervals are defined at local authority level and are not directly transferrable to the national time series we constructed from aggregated counts.<br><br>The SAMHI exposure is a composite measure constructed from multiple administrative indicators, and aggregated to the national level using weighted means – again, for descriptive purposes.<br>Given the heterogeneous nature of the underlying data sources, there is no single consistent sampling framework for deriving meaningful confidence intervals across indicators.<br><br>We do agree on the importance of transparency about heterogeneity across local authorities over time. The distribution of variables both within and between areas is shown in the spaghetti plots in Supplementary Material Figures 2-4.	

**Reviewer #2**	**Authors' response**	**Corrections**<br>Page and line numbers cited in this section correspond to the simple revised manuscript, in which tracked changes have been accepted. In this column, for the sake of clarity, new material is underlined.
1. The opening paragraph establishes the importance of neglect and the policy relevance of reducing statutory child protection involvement, but the flow of the introduction feels somewhat choppy. The manuscript moves quickly from prevalence and policy priorities to definitions of neglect, conceptual debates, social workers' perceptions, and then empirical antecedents of maltreatment. As written, the connection among these ideas is not always clear. For example, the sentence describing social workers' recognition of parental mental health and addiction, but limited recognition of poverty, feels somewhat abrupt in its current placement. Similarly, the transition to "These conceptual and practical challenges have prompted empirical research into the antecedents of different types of child maltreatment" is not fully developed. The authors should revise the introduction to more	Many thanks for this comment. We have now revised the introduction to improve the clarity and flow. We now more clearly articulate 1) why neglect may be considered conceptually distinct from other maltreatment types, with potentially different antecedents; 2) how recorded neglect in administrative data may reflect both underlying need and processes of service contact and identification. These two revisions clarify the rationale for undertaking comparative analyses of risk factors for neglect and other maltreatment types; 3) we summarise the comparative literature, highlight gaps, and outline our aims.	Pages 4-5, lines 118-143<br><br>However, critical scholarship cautions against treating different forms of maltreatment as equivalent. While abuse is characterised by acts of commission, neglect is generally defined by acts of omission – not what carers do, but what they fail to do, or what they are unable to provide. This distinction raises questions about capacity and intention. Neglect-related harm may be unintentional, arising from constrained caregiver capabilities linked to disadvantaged socioeconomic conditions and family stress.[10–12] The social determinants may be especially relevant to understanding neglect, relative to other maltreatment types. A systematic review of social workers' perceptions of neglect found widespread recognition of the contribution of parental mental health problems and addiction to neglect – though limited recognition of the role of poverty in structuring these factors.[13]<br><br>These conceptual differences may also have implications for how neglect is identified and recorded. Measurement is a fundamental challenge in child maltreatment research.[7] Definitions of abuse and neglect are neither fixed nor culturally neutral.[8] Understandings of neglect in particular have also evolved over time, shaped by societal expectations of good parenting.[9] Within child welfare systems, a determination of child neglect also relies on policy frameworks, professional norms and judgement, intervention thresholds and data collection practices, all of which may vary within and between local authorities, regions, and countries. Administrative data are more likely to record forms of adversity that are more visible, disclosed or surveilled. Families experiencing poverty and poor mental health may be more likely to face both caregiving challenges and heightened scrutiny from services. Together, these

clearly build the logic of the study: why neglect may differ conceptually from other maltreatment types, why social determinants such as poverty and adult mental health may be especially relevant to neglect, and why comparing neglect with other maltreatment categories is necessary.		processes may shape observed patterns of recorded neglect. There is a lack of clarity on whether social determinants of adversity are specifically associated with recorded neglect, or more generally with child protection involvement. This has implications for policy strategies to safely reduce rates of neglect.<br><br>Empirical research into the antecedents of different types of child maltreatment shows substantial overlap in risk factors by type.[14,15]<br><br>Page 5, lines 159-162<br><br>Longitudinal analyses remain relatively scarce, and few studies span the last decade – a period in the UK marked by austerity policies, tiered COVID-19 pandemic restrictions,[20] and the rising cost of living,[21] each with potential implications for caregiving capacity and service response.[22]<br><br>Page 5, lines 168-172<br><br>First, we assessed whether adult mental health was associated with CPPs due to neglect, in English local authorities, between 2014/15 and 2022/23, comparing results with other recorded maltreatment types and with care entry, a more acute intervention. Second, we assessed the associations of child poverty and employment with neglect and all other recorded maltreatment types.
2. The PPI section is a strength and provides valuable context for how lived experience informed the framing and interpretation of the study. However, the current wording feels somewhat informal and includes personal biographical details about one author and their family that may be unnecessary or potentially identifying in a peer-reviewed manuscript. The sentence about including a future digital story also reads as provisional and may not be appropriate for the final manuscript. I recommend revising this section to focus more broadly on how lived experience and public advisory input shaped the research question, interpretation, and dissemination.	Thank you, we have now revised the PPIE section to remove biographical detail from main manuscript.<br><br>We have retained a link to the author's digital story as a complementary output. We feel that this reflects best practice in transparent reporting of researcher positionality, and provides additional context on how lived experience informed the development of the research question.	Page 8, lines 286-287<br><br>The research question was developed by a care-experienced member of the research team. This digital story provides further context on how lived experience informed the research: https://youtu.be/2FxefRH57D0?si=qLVFXHTaW2YqvMbr

3. Although the authors use a longitudinal fixed-effects design, the study remains ecological and observational. Some wording implies stronger causal inference than the design can support, particularly language around "drivers," "determinants," and the estimated number of additional cases associated with exposure trends. The authors should more consistently frame findings as area-level associations and clarify that the counterfactual estimates are model-based projections rather than causal estimates of attributable burden.	Many thanks – we have now revised the language throughout to clarify the area-level associational nature of the estimates, and the need for caution in interpreting counterfactual estimates.	Page 3, lines 77-84<br><br>Rising adult mental ill health was associated with higher rates of neglect and emotional abuse CPPs at area-level, but confidence intervals included the null. Child poverty was positively associated with neglect CPPs: a one-percentage point increase was associated with around one additional CPP per 10,000 children (95% CI 0.2-2.2), equivalent to a model-estimated difference of over 30,000 plans over the study period. Higher employment was associated with fewer physical abuse CPPs, though confidence intervals included the null. Care entry was positively associated with mental ill health and child poverty, and negatively associated with employment.<br><br>Page 4, lines 110-111<br><br>Achieving this objective requires an understanding of the modifiable factors associated with variation in neglect.<br><br>Page7, lines 266-269<br><br>The resulting estimates represent model-predicted differences in CPPs associated with observed change in exposure over the study period, relative to the counterfactual scenario. They should not be interpreted as causal estimates of attributable burden.<br><br>Page 9, lines 330-332<br><br>Table 3 presents model-based estimates of the differences in the numbers of children placed on CPPs or entering care associated with observed trends in exposures between 2014/15 and 2022/23<br><br>Page 10, lines 339-340<br><br>Estimated differences in the numbers of children associated with observed trends in exposures<br><br>Page 10, lines 346-353<br><br>However, effect sizes were imprecise, with wide confidence intervals including zero. Accordingly, rising SAMHI scores over the study period were associated with an estimated difference of 44,659 additional neglect CPPs (95% CI -3,763 to 94,911), and a difference of 30,424 emotional abuse CPPs (95% CI -6,724 to 68,860), relative to a counterfactual scenario in which SAMHI had remained constant from 2014/15. There was little evidence of an association with physical abuse. Higher SAMHI was, however, associated with higher rates of children entering care (4.40, 95% CI 1.05 to 7.74), corresponding to an estimated difference of 37,307 care entries over the study period (95% CI 9,266 to 66,432).

		Page 10, lines 354-359<br><br>Child poverty showed a positive association with neglect CPPs. A one percentage point increase in child poverty was associated with approximately one neglect CPP per 10,000 children (1.22, 95% CI 0.22 to 2.23), equivalent to an estimated difference of 33,248 neglect CPPs overall (95% CI 5,635 to 60,057). Poverty was also positively associated with care entry rates, corresponding to an estimated difference of 16,594 care entries (1,887 to 30,783).<br><br>Pages 10-11, lines 360-366<br><br>Employment rates were not meaningfully associated with either neglect or emotional abuse CPPs. There was some evidence of an association with lower physical abuse CPP rates, but confidence intervals included the null (-0.10, 95% CI -0.20 to 0.00). Higher employment rates were also associated with lower care entry rates, with wide uncertainty (-0.17, 95% CI -0.33 to -0.01). Accordingly, overall improvements in employment over the study period were associated with an estimated difference of -2,178 physical abuse CPPs (95% CI -4,323 to 50), and -3,670 care entries (-7,119 to -88).<br><br>Page 11, lines 390-393<br><br>Among area-level social and economic factors, child poverty showed an association with higher rates of CPPs due to recorded neglect, with little evidence of comparable associations for recorded emotional and physical abuse.<br><br>Page 11, lines 198-400<br><br>Together, these findings suggest that the occurrence and identification of neglect may reflect broader social conditions and stressors potentially affecting parental caregiving capacity and service response.[37,38]<br><br>Page 12, lines 435-443<br><br>First, the ecological design means that findings reflect results at the local authority level, not the individual level. They cannot determine whether families of children being placed on a CPP are also experiencing worsening adult mental health, poverty, or employment changes occurring within their local authority. At the local authority level, the processes shaping exposures and covariates are less readily disentangled, contributing to wider uncertainty in adjusted estimates; exposures should be interpreted as contextual indicators of area-level mental health, economic conditions and labour market participation, rather than direct measures of parental mental health, family poverty or family employment.

		Page 13, lines 450-451<br><br>Second, our main exposure, the adapted SAMHI, may be a crude proxy for area-level parental mental health, the phenomenon of interest.<br><br>Page 14, lines 502-504<br><br>Finally, policymakers should consider our findings of a modest negative association of employment with care entry, alongside broader uncertain evidence of the effect of maternal employment.
4. The manuscript frames child poverty as having a specific association with neglect compared with other maltreatment types. However, it is not clear that the authors formally tested whether coefficients differed across maltreatment categories. Given overlapping confidence intervals across outcomes, the evidence for specificity should be interpreted cautiously. The authors should either formally test differences across outcomes or soften this language.	Thank you for this – we agree and have removed any language that could be interpreted as claiming that associations are outcome-specific.	Page 3, lines 78-79<br><br>Child poverty was positively associated with neglect CPPs.<br><br>Page 11, lines 390-393<br><br>Among area-level social and economic factors, child poverty showed an association with higher rates of CPPs due to recorded neglect, with little evidence of comparable associations for recorded emotional and physical abuse.<br><br>Page 12, line 421<br><br>The association with physical abuse is common<br><br>Page 17, lines 479-481<br><br>The suggestive association between child poverty and identified neglect, and the association with care entry, lends additional weight to calls for national anti-poverty and poverty-reduction strategies.
5. Several central findings, particularly those involving SAMHI and neglect or emotional abuse CPPs, have wide confidence intervals that include the null. Although the estimated effect sizes are meaningful, the uncertainty is substantial. The Discussion and Abstract should more clearly distinguish between robust findings and suggestive patterns. Relatedly, the abstract states that adult mental ill health was associated with substantially higher rates of neglect and emotional abuse CPPs, although confidence intervals spanned the null. This should be worded	Many thanks. We have now worded these results more cautiously to avoid overstating the strength of the evidence.	Page 3, lines 77-84<br><br>Results. Rising adult mental ill health was associated with higher rates of neglect and emotional abuse CPPs at area-level, but confidence intervals included the null. Child poverty was positively associated with neglect CPPs: a one-percentage point increase was associated with around one additional CPP per 10,000 children (95% CI 0.2-2.2), equivalent to a model-estimated difference of over 30,000 plans over the study period. Higher employment was associated with fewer physical abuse CPPs, though confidence intervals included the null. Care entry was positively associated with mental ill health and child poverty, and negatively associated with employment.

more cautiously so the abstract does not overstate the strength of evidence.		
6. Child Protection Plans reflect both underlying maltreatment risk and the functioning of the child protection system. This is especially important for neglect, which is more dependent on professional judgment, local thresholds, socioeconomic surveillance, and service availability than some other maltreatment categories. The manuscript would benefit from deeper consideration of whether observed associations reflect changes in neglect itself, changes in identification, or changes in administrative responses. This discussion should also consider whether changes in local thresholds, workforce capacity, funding cuts, inspection pressures, policy reforms, social care staffing shortages, or referral pathways may have influenced CPP rates independently of actual maltreatment need.	Thank you, we agree that this is an important point that we needed to engage with more fully, and carry through the paper from introduction to discussion.	Page 4, lines 132-141<br><br>Within child welfare systems, a determination of child neglect also relies on policy frameworks, professional norms and judgement, intervention thresholds and data collection practices, all of which may vary within and between local authorities, regions, and countries. Administrative data are more likely to record forms of adversity that are more visible, disclosed or surveilled. Families experiencing poverty and poor mental health may be more likely to face both caregiving challenges and heightened scrutiny from services. Together, these processes may shape observed patterns of recorded neglect. There is a lack of clarity on whether social determinants of adversity are specifically associated with recorded neglect, or more generally with child protection involvement. This has implications for policy strategies to safely reduce rates of neglect.<br><br>Page 11, lines 398-403<br><br>Together, these findings suggest that both the occurrence and recorded identification of neglect may reflect broader social conditions and stressors potentially affecting parental caregiving capacity and service response.[36,37] At the level of underlying need, socioeconomic conditions and material stress may affect parents' ability to cope and draw the notice of child protection systems.[38] Negative perceptions of parents in poverty,[38] and conditions of resource constraint may shape service delivery, affecting quality, workforce capacity, and thresholds for intervention.[39–41]<br><br>Page 12, lines 431-433<br><br>Relationships between social and health determinants and children's social care involvement are also likely to be shaped by complex socio-cultural contexts, policy environments, shock events and child protections systems.[48]<br><br>Page 12, lines 443-445<br><br>Moreover, our Children's Services outcomes reflect both underlying need and service response, further limiting our understanding of the mechanisms linking exposures and outcomes.
7. The use of linear fixed-effects models is reasonable and clearly presented, but the outcomes are rates derived from count data. The Poisson sensitivity analyses showed	Thank you for this question. We estimated both linear and Poisson fixed-effects models, but chose the linear specification for our main analysis, both because absolute measures are preferred for policy communication, and to better contextualise the policy relevance of our findings: in the paper, we translated the estimated	Page 11, lines 379-384<br><br>Sensitivity analyses using Poisson fixed effects models showed that the variance of the outcome exceeded the conditional mean within local authorities. Cluster-robust standard errors were used to account for clustering at the local authority level in the estimation of confidence intervals. Incidence rate ratios were

substantial overdispersion, which raises questions about the best modelling approach. The authors should provide a clearer justification for using linear fixed-effects models as the primary analytic strategy and explain whether alternative count models, such as negative binomial models, were considered.	exposure effects into the difference in the number of interventions associated with changes in exposure. When using linear fixed-effects models, the coefficient can be interpreted as a constant marginal effect in levels. Adding up effects across local authorities and over time to obtain the estimated total difference in social care interventions is therefore straightforward.<br><br>In contrast, Poisson models require us to reconstruct LA-specific fitted values, including fixed effects and baseline incidence, to obtain differences in expected counts, making aggregation less transparent. The linear fixed-effects models provide a more direct and transparent way of expressing results in terms of the difference in interventions.<br><br>Given that rates are derived from count data, we always intended to perform sensitivity analyses using count-data models. In the choice between Poisson fixed-effects and Negative Binomial fixed-effects, we prioritised the former. Wooldridge (1999) shows that the fixed-effects Poisson estimator consistently estimates the parameters of the conditional mean even when the data are overdispersed or not Poisson. We now have amended the supplementary materials to avoid implying that the count outcome is conditionally Poisson distributed.<br><br>Fixed-effects Negative Binomial models require stronger distributional assumptions, including restrictions linking the fixed-effects to the overdispersion structure in order for the likelihood to properly eliminate the fixed-effects (Guimaraes, 2008). It may therefore be more sensitive to misspecification, as well as suffering from a number of other issues (see, for instance, Wooldridge (2019), first post).<br><br>Diagnostics indicated substantial overdispersion even after accounting for LA and time fixed effects - this primarily affects efficiency and inference. We corrected the latter using cluster-robust standard errors at the fixed-effect (LA) level.<br><br>References<br><br>Guimaraes, P. (2008). The fixed effects negative binomial model revisited. Economics Letters, 99(1):63–66.	generally consistent in direction with the linear fixed effects results, although confidence intervals more frequently included the null. Results were most robust for SAMHI and care entry (Appendix 10).<br><br>Supplementary materials, page 7, lines 141-144<br><br>The dispersion statistic calculated from the Poisson fixed effects model residuals was 13.9, indicating that the variance exceeds the conditional mean within local authorities. Standard errors were obtained using cluster-robust variance estimation to account for overdispersion and serial correlation within local authorities.<br><br>Supplementary materials, page 2, lines 52-60<br><br>We estimate a Poisson regression model of the form<br>$\log(\mu_{it}) = \alpha E_{it} + \boldsymbol{x_{it}\beta} + \boldsymbol{d_t\gamma} + \delta_i + \log(N_{it}),$<br><br>where $\log(N_{it})$ is included as an offset, implying<br>$\log\left(\frac{\mu_{it}}{N_{it}}\right) = \alpha E_{it} + \boldsymbol{x_{it}\beta} + \boldsymbol{d_t\gamma} + \delta_i.$

	Wooldridge, J. M. (1999). Distribution-free estimation of some nonlinear panel data models. Journal of Econometrics, 90(1):77–97.<br><br>Wooldridge, J. M. (2019). Statalist forum discussion thread. Retrieved June 24, 2026 from https://www.statalist.org/forums/forum/general-stata-discussion/general/1508561-correcting-standard-errors-for-a-fixed-effects-poisson-model	
8. The estimated numbers of additional CPPs or care entries are useful for policy communication, but they depend heavily on the model specification and assumptions. The manuscript should be clearer that these estimates should not be interpreted as the number of cases that would necessarily have been prevented if poverty or adult mental ill health had remained unchanged.	Many thanks. We now caution the readers against causal interpretation, emphasise that the estimates are model-estimated differences rather than an attributable burden, and use the language of 'difference' rather than the more implicitly causal 'additional numbers' or 'fewer numbers'.	Page 3, lines 78-31<br><br>Child poverty was positively associated with neglect CPPs: a one-percentage point increase was associated with around one additional CPP per 10,000 children (95% CI 0.2-2.2), equivalent to a model-estimated difference of over 30,000 plans over the study period.<br><br>Page 7, lines 267-269<br><br>The resulting estimates represent model-predicted differences in CPPs associated with observed change in exposure over the study period, relative to the counterfactual scenario. They should not be interpreted as causal estimates of attributable burden.<br><br>Page 9, lines 326-330<br><br>Table 3 presents model-based estimates of the differences in the numbers of children placed on CPPs or entering care associated with observed trends in exposures between 2014/15 and 2022/23, compared to a counterfactual scenario in which each exposure (SAMHI, child poverty, employment) had remained at its 2014/15 LA-specific level, with other covariates maintained at their observed values over time.<br><br>Page 10, lines 339-340<br><br>Estimated differences in the numbers of children associated with observed trends in exposures<br><br>Page 10, lines 346-353<br><br>However, effect sizes were imprecise, with wide confidence intervals including zero. Accordingly, rising SAMHI scores over the study period were associated with an estimated difference of 44,659 additional neglect CPPs (95% CI -3,763 to 94,911), and a difference of 30,424 emotional abuse CPPs (95% CI -6,724 to 68,860), relative to a counterfactual scenario in which SAMHI had remained constant from 2014/15. There was little evidence of an association with physical abuse. Higher SAMHI was, however, associated with higher rates of children entering care (4.40, 95% CI 1.05 to 7.74), corresponding to an

		estimated difference of 37,307 care entries over the study period (95% CI 9,266 to 66,432).<br><br>Page 10, lines 354-359<br><br>Child poverty showed a positive association with neglect CPPs. A one percentage point increase in child poverty was associated with approximately one neglect CPP per 10,000 children (1.22, 95% CI 0.22 to 2.23), equivalent to an estimated difference of 33,248 neglect CPPs overall (95% CI 5,635 to 60,057). Poverty was also positively associated with care entry rates, corresponding to an estimated difference of 16,594 care entries (1,887 to 30,783).<br><br>Page 11, lines 364-366<br><br>Accordingly, overall improvements in employment over the study period were associated with an estimated difference of -2,178 physical abuse CPPs (95% CI -4,323 to 50), and -3,670 care entries (-7,119 to -88).
9. The manuscript would benefit from additional justification of the temporal structure of the models. Exposures and outcomes appear to be modelled within the same fiscal year, but it is plausible that changes in poverty, employment, or adult mental health may influence child protection outcomes with temporal lag. Some effects may emerge after months or years. Discussion of why contemporaneous associations were selected, and whether lagged models were explored, would strengthen the analytic rationale.	Many thanks. Existing evidence suggests that child protection interventions are responsive to relatively short-term changes in socioeconomic conditions. For example, quasi-experimental studies of labour shocks suggest that unemployment is associated with child maltreatment reports within the year, or with one-year lag structures (Brown and De Cao, 2018; Lindo et al., 2018). UK evidence indicates strong associations between area-level child poverty and care entry within the same year (Bennett et al., 2022). In our main analysis, we therefore focused on outcomes within the year. We have added a sentence to clarify.<br><br>However, we agree that child protection outcomes may also reflect conditions in previous years. We therefore conducted a sensitivity analysis, estimating a model using one-year lagged exposures. This trades off the first year of outcome data (2015) but extends series by an additional year (to 2024). Results were similar, with a coefficient of 5.0 additional child protection plans per 10,000 children associated with a one-standard deviation in the SAMHI in the year prior, controlling for prior-year confounders (95% CI -0.7-10.8). We have reported this sensitivity analysis in the manuscript, with results in the supplementary materials.<br><br>NB: in updating the code for the lagged analysis, we identified that the year	Page 7, lines 248-250<br><br>Since evidence suggests that child protection systems are relatively responsive to changes in socioeconomic conditions, we modelled associations within the same year.[33]<br><br>Page 7, lines 271-272<br><br>In sensitivity analyses, we assessed the temporal specification of the models by repeating analyses for the Neglect CPP outcome, using one-year lagged exposures and confounders.<br><br>Page 11 lines 368-369<br><br>Our sensitivity analysis using lagged exposure and confounders yielded similar findings, indicating robustness to alternative temporal specifications (Appendix 5).<br><br>Supplementary materials, page 5, lines 102-109<br><br>**Appendix 5. Linear fixed effects models for CPPs due to recorded neglect, with lagged exposures and confounders**<br>Table 2 shows results of our sensitivity analysis for the Neglect CPP outcome, using one-year lagged exposures. Results were similar to main model results, indicating robustness to temporal specification.

	variable for household structure data referred to mid-year. To ensure consistency with our convention of indexing all data by financial year (defined by the latter), we have now harmonised this variable and refitted the sensitivity analyses using additional confounders. Results are substantively similar.	
10. Adult mental health, child poverty, and employment are conceptually and statistically related constructs. The manuscript would benefit from additional discussion of potential multicollinearity among exposures and how this may influence coefficient stability and interpretation within the fixed-effects models.	Thank you, we agree that adult mental health, child poverty and employment are related, and a degree of collinearity between these variables is expected. In our fixed effects models, this is likely to increase standard errors and reduce precision of estimates, but it does not bias coefficients. Our adjustment strategy is guided by the DAG, and coefficients should be interpreted as conditional on the relevant adjustment sets. We have added a sentence to the limitations section to make this more explicit.	Page 12, lines 438-443<br><br>At the local authority level, the processes shaping exposures and covariates are less readily disentangled, contributing to wider uncertainty in adjusted estimates; exposures should be interpreted as contextual indicators of area-level mental health, economic conditions and labour market participation, rather than direct measures of parental mental health, family poverty or family employment.
11. The analyses assume independence across local authorities, yet neighbouring local authorities may share policy environments, economic conditions, or regional service structures. The manuscript would benefit from discussion of potential spatial dependence and whether spatial clustering or regional autocorrelation may influence estimates.	Thank you for this comment. We agree that neighbouring local authorities may share evolving policy environments, regional economic conditions and service structures. In a sensitivity analysis, we have additionally incorporated region fixed effects to account for shared time-invariant regional characteristics. Results were unchanged. We have also acknowledged the potential for residual spatial dependence in time-varying unobserved factors in the limitations section.	Page 7, lines 272-274<br><br>To account for potential shared regional trends in policy and economic conditions, we re-estimated the main model additionally incorporating region fixed effects.<br><br>Page 11, lines 369-370<br><br>Results were also robust to adjustment for region-level fixed effects (Appendix 6).<br><br>Page 13, lines 458-461<br><br>Third, neighbouring local authorities may share evolving policy environments, economic conditions and service structures. Although our sensitivity analysis using both local authority and region fixed effects suggests that this is unlikely to material affect our results, residual spatial dependence in time-varying unobserved factors may remain.<br><br>Supplementary materials, page 5, lines 110-116<br><br>**Appendix 6. Linear fixed effects models for CPPs due to recorded neglect, additionally incorporating region fixed effects.**<br>Table 3 shows results of analyses for the Neglect CPP outcome, with and without region fixed effects. The inclusion of region fixed effects does not materially change point estimates or inference, with only marginal changes in standard errors
12. Additional detail regarding the frequency and distribution of	Many thanks. To improve transparency, we have presented a table of suppressed values by outcome type in the appendix,	Page 5, lines 189-193

suppressed/imputed values across outcomes and years would improve transparency. Because low-count suppression may disproportionately affect certain maltreatment categories, especially sexual abuse CPPs, the potential impact of this imputation strategy on estimates should be discussed more explicitly. Although the random imputation of suppressed counts between one and five seems reasonable and sensitivity analyses are reassuring, the manuscript should more explicitly state how many values were suppressed by outcome type.	and added a summary sentence to the main manuscript.	Counts between one and five are suppressed in publicly available data. Suppression was rare for CPPs due to recorded Neglect and Emotional abuse (n=21), and common for less frequent physical abuse (n=245) and sexual abuse CPPs (n=490). Suppressed values were therefore imputed by drawing a random integer between one and five, and robustness to imputation strategy assessed in sensitivity analyses described below.<br><br>Supplementary materials, page 1, lines 2-8<br><br>**Appendix 1. Suppressed data**<br>Counts between 1 and 5 were suppressed in the publicly available data for confidentiality. Table 1 shows the count and proportion of local-authority-years for which data were supressed, by outcome. Suppressed counts were rare for Neglect and Emotional abuse CPPs, and common for less frequent physical and sexual abuse CPPs.
13. The area-level exposure measures may not capture the family-level mechanisms implied in parts of the interpretation. SAMHI captures area-level adult mental health, not specifically caregiver or parental mental health; employment is working-age population employment, not parent employment; and poverty is measured at the area level rather than the family level. The manuscript should more clearly acknowledge that these measures may function as broad area-level indicators rather than direct measures of caregiving capacity, caregiver employment conditions, or family economic hardship.	Thank you – we have now been much more explicit about this in the limitations section.	Page 12, lines 440-443<br><br>exposures should be interpreted as contextual indicators of area-level mental health, economic conditions and labour market participation, rather than direct measures of parental mental health, family poverty or family employment.
14. The appendix provides helpful detail, but because the adapted SAMHI is central to the analysis, the main text should briefly explain why the original index was modified and what indicator was removed.	Many thanks, and we agree. We have now given a brief explanation of why we modified the SAMHI in the main manuscript.	Page 6, lines 199-214<br><br>Our primary exposure was an adapted version of the Small Area Mental Health Index (SAMHI), a composite annual measure of population mental health for each lower super output area (LSOA) in England, available from the Place Based Longitudinal Data Resource.[27] The original SAMHI incorporated data on mental health-related hospital attendances, which showed major post-2019/20 discontinuities consistent

		with disrupted access following pandemic restrictions (Appendix 1). Our adapted SAMHI therefore excludes this indicator, combining data on remaining indicators: antidepressant prescriptions; percentage of adult patients with new diagnoses of depression; and Disability Living Allowance and Personal Independence Payments for reasons of mental ill health and learning difficulties. These indicators were rescaled to have a mean of 0 and standard deviation of 1, and maximum likelihood factor analysis was applied to compute a single factor score based on intercorrelations among the components. We validated this approach by using it to reconstruct the SAMHI from all four original indicators and comparing it with published SAMHI data, finding close correspondence (r=0.96). We aggregated the adapted SAMHI to the upper-tier local authority level, weighting by LSOA population, and re-standardised the resulting scores such that the mean across all local authorities and years was 0 with a standard deviation of 1. Higher scores represent worse mental health.[28]
15. The manuscript should specify whether SAMHI was standardized across all local authorities and years combined or within each year, as this affects interpretation of a one-standard-deviation increase.	Thank you. SAMHI was standardised across all local authority-years combined, ensuring that coefficients represent associations for a one standard deviation increase in the pooled distribution over the full study period. We have specified this in the methods section and hope it will be clear to readers.	Page 6, lines 211-214<br><br>We aggregated the adapted SAMHI to the upper-tier local authority level, weighting by LSOA population, and re-standardised the resulting scores such that the mean **across all local authorities and years** was 0 with a standard deviation of 1. Higher scores represent worse mental health.[28]
16. Table 3 notes that highlighted cells denote associations of interest based on effect size and confidence intervals, but no formal cutoff was used. This could unintentionally guide readers toward selected interpretations. Consider removing the highlighting or defining objective criteria.	Thank you and agreed, we have now removed the shading and notes.	Page 10, lines 339-341
17. The manuscript should consistently distinguish among “neglect,” “recorded neglect,” and “CPPs due to neglect.” This distinction is important because the study measures administrative child protection outcomes, not maltreatment prevalence directly.	Thank you, and we agree. We have made changes throughout the manuscript to preserve the distinction.	Page 3, lines 85-86<br><br>**Conclusions:** Recorded neglect and care entry appear particularly sensitive to wider social conditions affecting caregiving capacity and service response.<br><br>Page 5, lines 168-172<br><br>In this study, we aimed to fill these gaps. First, we assessed whether adult mental health was associated with CPPs due to recorded neglect, in English local authorities, between 2014/15 and 2022/23, comparing results with other recorded maltreatment types and with care entry, a more acute intervention. Second, we assessed the associations of child poverty and

		employment with recorded neglect and all other maltreatment types.<br><br>Page 7, lines 241-243<br><br>Second, we fitted two-way linear fixed effects models to estimate within-local authority associations between our main exposure, the adapted SAMHI, and our main outcome, the rate of new CPPs due to recorded neglect, between 2014/15 and 2022/23.<br><br>Page 7, lines 253-255<br><br>Third, we fitted similar models to estimate associations of secondary exposures, child poverty and employment rates, with recorded neglect and all other maltreatment types, controlling for their respective confounders<br><br>Page 8, lines 304-306<br><br>Across 147 local authorities, between 2014/15 and 2022/23, rates of new CPPs were highest for recorded neglect, followed by emotional and physical abuse; recorded sexual abuse remained rare.<br><br>Page 10, lines 343-346<br><br>Between 2014/15 and 2022/23, within English local authorities and controlling for employment and child poverty rates, higher SAMHI scores were associated with higher rates of children being placed on CPPs due to recorded neglect and emotional abuse<br><br>Page 11, lines 387-394<br><br>In this longitudinal ecological analysis of English local authorities (2014/15-2022/23), worsening adult mental health was associated with higher rates of children being placed on CPPs due to recorded neglect and emotional abuse, but not physical abuse, after controlling for key confounders – but substantial uncertainty limits strong inference. Among area-level social and economic factors, child poverty showed an association with higher rates of CPPs due to recorded neglect, with little evidence of comparable associations for emotional and physical abuse. There was weak evidence that higher employment rates were associated with lower rates of CPPs due to recorded physical abuse only, again with wide uncertainty<br><br>Page 11, lines 402-403<br><br>Negative perceptions of parents in poverty,[39] and conditions of resource constraint may shape service delivery, affecting quality, workforce capacity, and thresholds for intervention.[40–42]<br><br>Pages 11-12, lines 404-409

		Our results broadly align with the wider literature, with some divergences. Our finding of potential positive associations of mental ill health with both recorded neglect and emotional abuse is consistent with systematic review evidence identifying parental mental ill health as a shared risk factor across abuse types – though our null finding for recorded physical abuse diverges from these reviews.[14,16] In our analyses, associations appeared larger for recorded neglect than for recorded emotional abuse<br><br>Page 12, lines 412-215<br><br>Similarly, our findings of a positive association between child poverty and recorded neglect aligns with systematic review evidence – but we find no clear associations with recorded emotional and physical abuse where reviews report positive associations of comparable magnitude.[14,16]<br><br>Page 13, lines 479-481<br><br>The suggestive association between child poverty and recorded neglect, and the association with care entry, lends additional weight to calls for national anti-poverty and poverty-reduction strategies.<br><br>Page 14, lines 510-511<br><br>Recorded neglect and care entry appear particularly sensitive to broader social and economic stressors; emotional and physical abuse show more mixed patterns.
18. The study period spans COVID-19 disruptions, which likely affected both exposure measurement and child protection processes. The manuscript would benefit from a clearer explanation of how year fixed effects and the adapted SAMHI address these disruptions.	Many thanks. In the main manuscript, we have now clarified how we addressed potential COVID-19-related disruption, including mitigation of exposure measurement issues through the adapted SAMHI, and the role of year fixed effects in accounting for national time-specific shocks affecting child protection outcomes.	Page 6, lines 199-207<br><br>Our primary exposure was an adapted version of the Small Area Mental Health Index (SAMHI), a composite annual measure of population mental health for each lower super output area (LSOA) in England, available from the Place Based Longitudinal Data Resource.[27] The original SAMHI incorporated data on mental health-related hospital attendances, which showed major post-2019/20 discontinuities consistent with disrupted access following pandemic restrictions (Appendix 1). Our adapted SAMHI therefore excludes this indicator, combining data on remaining indicators: antidepressant prescriptions; percentage of adult patients with new diagnoses of depression; and Disability Living Allowance and Personal Independence Payments for reasons of mental ill health and learning difficulties.<br><br>Page 7, lines 246-247<br><br>Year fixed effects capture changes occurring across all local authorities in each year, including the Covid-19 pandemic and associated disruptions to economic conditions and children's social care processes.

19. The Methods mention that boundary changes were harmonized where possible, but additional detail would improve reproducibility.	Thank you, we agree. We have added a sentence to the methods section. We have also made all raw data and code available to support reproducibility (per comment 22).	Page 5, lines 175-179<br><br>During this time, some local authorities underwent boundary changes. Where local authorities split cleanly (Northamptonshire into North and West Northamptonshire from 2021/22, Cumbria into Cumberland and Westmorland and Furness from 2023/24), we harmonised the data, retaining the pre-split boundary throughout the study period.<br><br>Page 8, line 287<br><br>Data and code are available at: https://drive.google.com/drive/folders/1VsgNEOU1diGNQVIsOAi-I4eXIrALZS2z?usp=sharing
20. The policy section is compelling, but some recommendations imply that the observed associations directly identify intervention targets. The authors should ensure that policy implications remain proportionate to the ecological evidence.	Many thanks – we have edited this section to temper the language, and make it clear that recommendations derive from the wider evidence base alongside our findings.	Page 13, lines 472-489<br><br>*Implications for policy*<br>Our findings have a number of implications for population-level policy. Together with the broader evidence base on parental mental ill health as a common and salient factor in children's social care involvement,[49,50] they suggest that supporting adult mental health is not only a health priority, but also a child protection imperative. Timely, accessible mental health support for parents, alongside cross-sector collaboration across health and social care, adult and children's services, may help address conditions associated with identified neglect, emotional abuse and care entry.<br>The suggestive association between child poverty and recorded neglect, and the association with care entry, lends additional weight to calls for national anti-poverty and poverty-reduction strategies. There is increasing evidence that policies that increase household income and buffer against economic shocks can help alleviate the social conditions that drive neglect and poor subsequent adolescent health outcomes.[37,51,52] For national policymakers, a first step might be setting child poverty targets.[53] At a local level, practitioners should be supported to recognise child poverty as a modifiable factor associated with neglect and care entry,[10,18] and address it where possible.[54] Given the conceptual and operational challenges in defining and responding to neglect, greater consistency in recording families' specific socioeconomic circumstances and health needs may help direct appropriate support to those experiencing adversity associated with neglect.[50]
21. The heading appears to state "2014/15-20219/20," which should likely be corrected to "2014/15-2019/20."	Thank you, we have now corrected the typo.	Page 11, lines 374-377<br><br>Restricting the dataset to years 2014/15 to 2019/20 to control for additional confounders resulted in some attenuation of the association between SAMHI and both emotional abuse CPPs (from 4.32 to 3.63) and

		care entry (from 4.58 to 3.81), with wide uncertainty and overlapping confidence intervals.
22. The manuscript states that all data are aggregate and publicly available, and that code is available from the corresponding author on request. Given BMJ Open's emphasis on transparency, the authors should consider providing direct links to the datasets and making analytic code available in a public repository or explaining why this is not possible.	Thank you. In this revision we have shared a link to all data, and to scripts, which include links to raw data sources.<br><br>The code was written by the joint first authors, with code check by the senior author. We prioritised clarity and reproducibility over conciseness.	Page 8, line 287<br><br>Data and code are available at: https://drive.google.com/drive/folders/1VsgNEOU1diGNQVIsOAi-I4eXIrALZS2z?usp=sharing
23. The authors state that confounders were identified using a DAG, but the rationale for included and excluded variables is not fully explained. A clearer explanation of the assumed relationships among SAMHI, poverty, employment, and CPP outcomes would help readers evaluate the adjustment strategy.	Many thanks for this. We have now expanded our description of the DAG to more clearly articulate assumed relationships among the variables used to guide confounder adjustment. In this framework, child poverty and employment are specified as determinants of both mental ill health and child protection outcomes, and are therefore included in our main models. Additional identified confounders identified in the a priori DAG informed our choice of sensitivity analyses. This supports transparent assessment of our adjustment strategy and the potential for residual confounding.	Page 6, lines 224-231<br><br>We used a Directed Acyclic Graph (DAG, figure 1) to specify a priori assumptions about relationships between area-level variables, and to guide confounder selection for all analyses. It illustrates the focal relationship between adult mental ill health (the primary exposure) and statutory Children's Services interventions (including CPPs due to recorded neglect, the primary outcome). It shows theorised confounders – including ethnic composition, household structure, employment and child poverty, ordered conceptually from more distal to more proximal determinants. And it shows theorised mediators through which the exposure may operate.
24. The manuscript notes the ecological design, but it could more explicitly state that the analysis cannot determine whether children entering CPPs are in the same families or areas experiencing worsening adult mental health, poverty, or employment changes. This limits conclusions about mechanisms linking social determinants to neglect.	Thank you, we have now been much more explicit about the ecological design and its drawbacks in our limitations section.	Page 12, lines 435-447<br><br>This study has important limitations. First, the ecological design means that findings reflect results at the local authority level, not the individual level. They cannot determine whether families of children being placed on a CPP are also experiencing worsening adult mental health, poverty, or employment changes occurring within their local authority. This limits our understanding of the mechanisms linking exposures and outcomes. Exposures should be interpreted as contextual indicators rather than direct measures of family experience. Nevertheless, the local authority is a policy-relevant unit of analysis, and our study captures complete data for a critical period in England, spanning post-recession austerity years, pandemic disruption, and a period of rising living costs.
25. Fixed effects handle stable differences across local authorities, but not changing time-varying contextual factors such as local service funding, housing insecurity, migration, school closures, social care staffing shortages, or	Many thanks for this comment, and we agree about the limitations of our fixed effects models.<br><br>Some of the service factors you raise (Children's Services capacity) we conceived as likely mediators, since central government funding allocations to local authorities are determined in part by deprivation and levels of need. We were	Page 13, lines 462-465<br><br>Fourth, sensitivity analyses adjusting for additional confounders showed minimal attenuation of estimates, but some of confounders were only available at regional level or were imperfect proxies for DAG variables. Residual confounding due to these and other time-varying factors (e.g. housing insecurity, school closures), is possible.

changes in referral practices. These sources of residual time-varying confounding should be acknowledged more explicitly.	primarily interested in the more upstream social and economic conditions.<br><br>However, we recognise that some of the factors you raise could certainly introduce residual time varying confounding, and we have noted them in the limitations section.	

VERSION 2 - REVIEW

Reviewer 1

Name Kelson, Zoe

Affiliation University of Exeter, Mathematics

Date 08-Jul-2026

COI

Thanks to the authors for their responses, providing their rationale, undertaking additional analyses, and revising the article where required.
