# Supplementary material for: Is neglect different? Social determinants of child protection plans by recorded maltreatment type in England: a longitudinal ecological study, 2014/2015–2022/2023
Source: BMJ Open. 2026 Aug 5;16(8):e119211. doi: 10.1136/bmjopen-2026-119211 (PMC13448618; doi:10.1136/bmjopen-2026-119211)
Supplement: Supplementary file 2 [file bmjopen-16-8-s001.docx]

**Supplementary materials**

**Appendix 1. Suppressed data**

Counts between 1 and 5 were suppressed in the publicly available data for confidentiality. Table 1 shows the count and proportion of local-authority-years for which data were supressed, by outcome. Suppressed counts were rare for Neglect and Emotional abuse CPPs, and common for less frequent physical and sexual abuse CPPs.

Table 1. Count of local-authority-years for which CPP data were suppressed by recorded child maltreatment type

| **Outcome** | **Supressed (count)** | **Proportion suppressed (%)** |
| --- | --- | --- |
| Neglect CPP | 21 | 0.01 |
| Emotional abuse CPP | 21 | 0.01 |
| Physical abuse CPP | 245 | 13.4 |
| Sexual abuse CPP | 490 | 26.9 |

**Appendix 2. Trends in original SAMHI indicators**

The original SAMHI combines data on: antidepressant prescriptions; percentage of adult patients with new diagnoses of depression; Disability Living Allowance and Personal Independence Payments for reasons of mental ill health and learning difficulties; and mental health-related hospital attendances.^1^ Local authority trends in the original SAMHI showed sudden decreases in the first year of the COVID-19 pandemic, warranting further exploration. Trends in the underlying indicators showed major post-2019/20 discontinuities for one single indicator, mental health-related hospital admissions, consistent with disrupted access following pandemic restrictions (Figure 1).^2^ We therefore computed an adapted SAMHI, excluding the hospital admissions indicator: remaining indicators were rescaled to have a mean of 0 and standard deviation of 1, and maximum likelihood factor analysis was applied to compute a single factor score based on intercorrelations among the components. We validated this approach by reconstructing a 4-component SAMHI and comparing it with the original SAMHI, finding close correspondence (r=0.96).

Figure 1. Trends in original SAMHI indicators.

**Appendix 3. Model formulae**

**Two-way linear fixed-effects model**

Let $i=1,\ldots,n$ index LAs and $t\in\{2014/15,\ldots,2022/23\}$fiscal years. We estimate the linear model

$$\begin{matrix} & y_{it}=\alpha E_{it}+\boldsymbol{x}_{\boldsymbol{it}}\boldsymbol{\beta}+\boldsymbol{d}_{\boldsymbol{t}}\boldsymbol{\gamma}+\delta_{i}+\varepsilon_{it}, & & \text{(1)} \end{matrix}$$

where $y_{it}$ is the outcome, $E_{it}$ is the exposure of interest, $\boldsymbol{x}_{\boldsymbol{it}}$ is a row vector of time-varying confounders, $\boldsymbol{d}_{\boldsymbol{t}}$ is a row vector of fiscal-year dummies, with 2014/15 as the reference year, $\delta_{i}$ is a LA-specific fixed effect capturing all time-invariant unobserved heterogeneity, and $\varepsilon_{it}$ is an idiosyncratic error term.

The within (fixed-effects) estimator transforms each variable by subtracting the LA-specific mean, effectively eliminating $\delta_{i}$ and controlling for all time-invariant LA-specific characteristics.

We estimate model (1) under different specifications of the exposure $E_{it}$, confounders $\boldsymbol{x}_{\boldsymbol{it}}$ and outcome $y_{it}$:

1. Exposure: parental mental health. Time-varying confounders: child poverty and employment rates. Outcome: rates per 10,000 children of new CPPs due to neglect; alternative outcomes: rates per 10,000 children of new CPPs due to emotional abuse, rates per 10,000 children of new CPPs due to physical abuse, and rates per 10,000 children of new cases of children entering care.
2. Exposure: child poverty. Time-varying confounders: employment rates. Outcomes as above.
3. Exposure: employment rates. Time-varying confounders: none. Outcomes as above.
4. For analyses restricted to $t\in\{2014/15,\ldots,2019/20\}$, exposures as above with additional time-varying confounders for ethnic composition and household structure in $\boldsymbol{x}_{\boldsymbol{it}}$**.**

**Poisson fixed-effects model**

Let $\tilde{y}_{it}$ denote counts of events (new cases of CPPs due to neglect, new cases of CPPs due to emotional abuse, new cases of CPPs due to physical abuse, or new cases of children entering care), and $N_{it}$ the population of children. Define the conditional mean

$$\mu_{it}\equiv E[\tilde{y}_{it}\mid E_{it},\boldsymbol{x}_{\boldsymbol{it}},\delta_{i},\boldsymbol{d}_{\boldsymbol{t}},N_{it}].$$

We estimate a Poisson regression model of the form

$$\begin{matrix} & \log(\mu_{it})=\alpha E_{it}+\boldsymbol{x}_{\boldsymbol{it}}\boldsymbol{\beta}+\boldsymbol{d}_{\boldsymbol{t}}\boldsymbol{\gamma}+\delta_{i}+\log(N_{it}), & & \text{(2)} \end{matrix}$$

where $\log(N_{it})$is included as an offset, implying

$$\log\left( \frac{\mu_{it}}{N_{it}} \right)=\alpha E_{it}+\boldsymbol{x}_{\boldsymbol{it}}\boldsymbol{\beta}+\boldsymbol{d}_{\boldsymbol{t}}\boldsymbol{\gamma}+\delta_{i}.$$

The estimated IRR of the exposure is $\exp(\hat{\alpha})$, representing the multiplicative change in the rate of new cases associated with a one-unit increase in $E_{it}$, holding constant time-varying confounders and fixed-effects. The exposure and confounders are defined analogously to scenarios 1–4 of the linear model (1).

**Model-predicted outcomes under counterfactual exposures**

Using the estimates from model (1), for each LA $i$and fiscal year $t\in\{2014/15,\ldots,2022/23\}$, fitted values can be constructed under the observed explanatory variables

$$\hat{y}_{it}^{ob}=\hat{\alpha}E_{it}+\boldsymbol{x}_{\boldsymbol{it}}\hat{\boldsymbol{\beta}}+\hat{\gamma}_{t},$$

and under a counterfactual scenario in which exposure is fixed at its 2014/15 value

$$\hat{y}_{it}^{co}=\hat{\alpha}E_{i,2014/15}+\boldsymbol{x}_{\boldsymbol{it}}\hat{\boldsymbol{\beta}}+\hat{\gamma}_{t},$$

where $\hat{\gamma}_{t}\in\hat{\boldsymbol{\gamma}}=(\hat{\gamma}_{2014/15},\ldots,\hat{\gamma}_{2022/23})$ and $\hat{\gamma}_{2014/15}=0$ as 2014/15 is the reference year.

The total model-predicted additional cases in the UK attributable to changes in exposure are obtained by summing, over all LAs and fiscal years, the difference between observed and counterfactual fitted values, scaled by the population of children

$$\begin{matrix} & \sum_{i} \sum_{t} \left( \hat{y}_{it}^{ob}-\hat{y}_{it}^{co} \right)\frac{N_{it}}{10,000}. & & \text{(3)} \end{matrix}$$

Fixed effects are absorbed by the within-transformation and are not explicitly included when constructing fitted values. They would enter both the observed and counterfactual predictions identically and therefore would drop out when taking differences in predicted outcomes.

To construct 95% confidence intervals, we draw 1,000 sets of coefficients for prediction based on model (1) from the multivariate normal distribution $\mathcal{N}(\hat{\boldsymbol{\iota}},{\hat{\boldsymbol{\Sigma}}}_{\boldsymbol{\iota}})$, with mean $\hat{\boldsymbol{\iota}}=(\hat{\alpha},\hat{\boldsymbol{\beta}},\hat{\boldsymbol{\gamma}})$ and variance–covariance matrix ${\hat{\boldsymbol{\Sigma}}}_{\boldsymbol{\iota}}$. Then, we recompute the total model-predicted additional cases for each draw, and take the empirical 2.5th and 97.5th percentiles across simulations.

Given that all covariates other than exposure are held fixed at their observed values in both scenarios, their contributions cancel in the difference. Therefore, the difference between observed and counterfactual predicted counts reduces to $\sum_{i} \sum_{t} \hat{\alpha}\left( E_{it}-E_{i,2014/15} \right)\frac{N_{it}}{10,000}.$

**Appendix 4. Spaghetti plots of trends in outcomes, exposure and covariates.**

Figures 2-4 show trends in outcomes, primary exposure (comparing original and adapted SAMHI), and secondary exposures, respectively. Grey lines represent individual local authorities, coloured lines the national mean.

Figure 2. Trends in Child Protection Plans across 147 English local authorities, 2014/15-2022/23.

Figure 3. Trends in the original and adapted SAMHI across 147 English local authorities, 2014/15-2022/23.

Figure 4. Trends in major covariates across 147 English local authorities, 2014/15-2022/23.

**Appendix 5. Linear fixed effects models for CPPs due to recorded neglect, with lagged exposures and confounders**

Table 2 shows results of our sensitivity analysis for the Neglect CPP outcome, using one-year lagged exposures. Results were similar to main model results, indicating robustness to temporal specification.

Table 2. Within-local authority associations of one-year-lagged adult mental health, child poverty, and employment, with CPPs due to recorded neglect, 2015/16-2023/24

| **Outcomes** | **Annual change in rate per 10,000 children (95% CI) over the study period** | | |
| --- | --- | --- | --- |
|  | 1. For a SD increase in the lagged SAMHI, controlling for lagged child poverty and employment | 2. For a %-point increase in lagged child poverty, controlling for lagged employment | 3. For a %-point increase in lagged employment. |
| Neglect CPP | 5.05 (-0.68, 10.78) | 0.85 (0.01, 1.71) | -0.06 (-0.35, 0.24) |

**Appendix 6. Linear fixed effects models for CPPs due to recorded neglect, additionally incorporating region fixed effects.**

Table 3 shows results of analyses for the Neglect CPP outcome, with and without region fixed effects. The inclusion of region fixed effects does not materially change point estimates or inference, with only marginal changes in standard errors

Table 3. Within-local authority associations of adult mental health, child poverty, and employment, with CPPs due to recorded neglect, with and without region foxed effects, 2014/15-2022/23

| **Neglect CPP outcome** | **Annual change in rate per 10,000 children (95% CI) over the study period** | | |
| --- | --- | --- | --- |
|  | 1. For a SD increase in the lagged SAMHI, controlling for child poverty and employment | 2. For a %-point increase in lagged child poverty, controlling for employment | 3. For a %-point increase in employment. |
| No region fixed effects | 5.28 (-0.49, 11.04) | 1.22 (0.22, 2.23) | -0.04 (-0.34, 0.27) |
| Region fixed effects | 5.28 (-0.59, 11.16) | 1.22 (0.20, 2.25) | -0.04 (-0.35, 0.27) |

**Appendix 7. Linear fixed effects models for CPPs due to recorded sexual abuse (low count outcome), and CPPs due to both recorded physical and sexual abuse (combined outcome)**

The sexual abuse CPP outcome was excluded from main analyses due to very low counts, high levels of data suppression, and limited within–local authority variation. Results are presented in Table 4 for completeness. In a sensitivity analysis, we combined sexual and physical abuse CPPs.

Table 4. Within-local authority associations of adult mental health, child poverty, and employment, with CPPs due to recorded sexual abuse, and CPPs due to both recorded sexual and physical abuse, 2014/15-2022/23

| **Outcomes** | **Annual change in rate per 10,000 children (95% CI) over the study period** | | |
| --- | --- | --- | --- |
|  | 1. For a SD increase in the SAMHI, controlling for child poverty and employment | 2. For a %-point increase in child poverty, controlling for employment | 3. For a %-point increase in employment. |
| Sexual abuse CPP | -0.90 (-1.46, -0.34) | 0.03 (-0.07, 0.12) | -0.04 (-0.08, -0.00) |
| Sexual and physical abuse CPP | -2.38 (-4.50, -0.27) | -0.15 (-0.26, -0.03) | -0.14 (-0.26, -0.03) |

**Appendix 8. Sensitivity analyses controlling for additional confounders**

Table 5 shows the results of sensitivity analyses restricted to years for which additional household and migration confounders are available. “Main” adjustment refers to the covariates used in our main models. “Additional” adjustment includes these plus: net international migration (local authority level), proportion of lone parent households (regional level), and proportion of households with 3+ dependent children (regional level).

Table 5. Within-local authority associations of adult mental health, child poverty, and employment, with children’s social care outcomes, 2014/15-20219/20, by confounder adjustment set.

| **Outcomes** | **Annual change in rate per 10,000 children (95% CI), 2014/15 to 2019/20** | | | | | |
| --- | --- | --- | --- | --- | --- | --- |
|  | For a SD increase in the SAMHI | | For a %-point increase in child poverty | | For a %-point increase in employment | |
| Confounder adjustment | Main | Additional | Basic | Additional | Basic | Additional |
| Neglect CPP | 6.88  (-0.48, 14.23) | 6.84  (-0.75, 14.43) | 1.77  (0.86, 2.67) | 1.70  (0.78, 2.63) | 0.18  (-0.24, 0.60) | 0.20  (-0.21, 0.62) |
| Emotional abuse CPP | 4.32  (-1.89, 10.53) | 3.63  (-2.84, 10.10) | 0.83  (0.01, 1.66) | 0.81  (-0.01, 1.63) | 0.11  (-0.24, 0.47) | 0.12  (-0.22, 0.47) |
| Physical abuse CPP | -2.70  (-5.55, 0.16) | -2.89  (-5.85, 0.08) | -0.16  (-0.48, 0.16) | -0.17  (-0.50, 0.16) | -0.17  (-0.31, -0.03) | -0.16  (-0.30, -0.03) |
| Care entry | 4.58  (-0.61, 9.76) | 3.81  (-1.54, 9.16) | 0.77  (0.16, 1.38) | 0.69  (0.10, 1.29) | 0.04  (-0.21, 0.29) | 0.06  (-0.19, 0.31) |

**Appendix 9. Sensitivity analyses using extreme imputation values**

Table 6. Within-local authority associations of adult mental health with children’s social care outcomes, 2014/15-2022/23, under different imputation strategies

| **Outcomes** | **Annual change in rate per 10,000 children (95% CI) for a SD increase in the SAMHI, controlling for child poverty and employment, 2014/15 to 2022/23** | | |
| --- | --- | --- | --- |
|  | Main models with random imputation | Imputation of minimum value | Imputation of maximum value |
| Neglect CPP | 5.28 (-0.49, 11.04) | 5.29 (-0.48, 11.06) | 5.24 (-0.52, 11.00) |
| Emotional abuse CPP | 3.60 (-0.82, 8.02) | 3.59 (-0.83, 8.02) | 3.59 (-0.84, 8.01) |
| Physical abuse CPP | -1.48 (-3.35, 0.38) | -1.46 (-3.35, 0.43) | -1.42 (-3.29, 0.42) |
| Care entry | 4.40 (1.05, 7.74) | 4.40 (1.06, 7.74) | 4.40 (1.06, 7.74) |

**Appendix 10. Sensitivity analyses using Poisson fixed effects models**

Figure 5 shows the distribution of within-local authority variance-to-mean ratios for the main outcome (annual counts of children placed on a CPP due to neglect). Ratios greater than one are suggestive of overdispersion. The dispersion statistic calculated from the Poisson fixed effects model residuals was 13.9, indicating that the variance exceeds the conditional mean within local authorities. Standard errors were obtained using cluster-robust variance estimation to account for overdispersion and serial correlation within local authorities.

Figure 5. Distribution of within-local authority variance-to-mean ratios for Neglect CPP counts

Poisson fixed effects model results are presented in table 6. Estimated incidence rate ratios are generally consistent in direction with the linear fixed effects models, although confidence intervals more frequently included the null. Primary inference is based on the linear fixed effects results.

Table 7. Incidence rate ratios from Poisson fixed effects models showing within-local authority associations of adult mental health, child poverty, and employment with children’s social care outcomes, 2014/15–2022/23.

| **Outcomes** | **Incidence rate ratios (95% CI) showing the multiplicative change in the rate of the outcome** | | |
| --- | --- | --- | --- |
|  | 1. For a SD increase in the SAMHI, controlling for child poverty and employment | 2. For a %-point increase in child poverty, controlling for employment | 3. For a %-point increase in employment. |
| Neglect CPP | 1.16 (0.95, 1.41) | 1.02 (0.99, 1.05) | 1.00 (0.99, 1.01) |
| Emotional abuse CPP | 1.11 (0.89, 1.39) | 1.01 (0.98, 1.04) | 1.00 (0.99, 1.01) |
| Physical abuse CPP | 0.75 (0.53, 1.07) | 1.02 (0.98, 1.07) | 0.99 (0.97, 1.01) |
| Care entry | 1.21 (1.08, 1.35) | 1.01 (0.99, 1.03) | 1.00 (0.99, 1.00) |

**Appendix references**

1	University of Liverpool. Place-based Longitudinal Data Resource. 2021. https://pldr.org/ (accessed June 16, 2020).

2	Bonello F, Zammit D, Grech A, Camilleri V, Cremona R. Effect of COVID-19 pandemic on mental health hospital admissions: comparative population-based study. *BJPsych open* 2021; **7**: e141.
